# Deciphering the etiology and role in oncogenic transformation of the CpG island methylator phenotype: a pan-cancer analysis

**DOI:** 10.1093/bib/bbab610

**Published:** 2022-02-02

**Authors:** Josephine Yates, Valentina Boeva

**Affiliations:** Institute for Machine Learning, Department of Computer Science, ETH Zürich, Zurich 8092, Switzerland; Institute for Machine Learning, Department of Computer Science, ETH Zürich, Zurich 8092, Switzerland; Swiss Institute for Bioinformatics (SIB), Zürich, Switzerland; Cochin Institute, Inserm U1016, CNRS UMR 8104, Paris Descartes University UMR-S1016, Paris 75014, France

**Keywords:** cancer, DNA methylation, CIMP, CpG island methylator phenotype, prognosis, genomic drivers

## Abstract

Numerous cancer types have shown to present hypermethylation of CpG islands, also known as a CpG island methylator phenotype (CIMP), often associated with survival variation. Despite extensive research on CIMP, the etiology of this variability remains elusive, possibly due to lack of consistency in defining CIMP. In this work, we utilize a pan-cancer approach to further explore CIMP, focusing on 26 cancer types profiled in the Cancer Genome Atlas (TCGA). We defined CIMP systematically and agnostically, discarding any effects associated with age, gender or tumor purity. We then clustered samples based on their most variable DNA methylation values and analyzed resulting patient groups. Our results confirmed the existence of CIMP in 19 cancers, including gliomas and colorectal cancer. We further showed that CIMP was associated with survival differences in eight cancer types and, in five, represented a prognostic biomarker independent of clinical factors. By analyzing genetic and transcriptomic data, we further uncovered potential drivers of CIMP and classified them in four categories: mutations in genes directly involved in DNA demethylation; mutations in histone methyltransferases; mutations in genes not involved in methylation turnover, such as *KRAS* and *BRAF*; and microsatellite instability. Among the 19 CIMP-positive cancers, very few shared potential driver events, and those drivers were only *IDH1* and *SETD2* mutations. Finally, we found that CIMP was strongly correlated with tumor microenvironment characteristics, such as lymphocyte infiltration. Overall, our results indicate that CIMP does not exhibit a pan-cancer manifestation; rather, general dysregulation of CpG DNA methylation is caused by heterogeneous mechanisms.

## Introduction

DNA methylation has been shown to play an essential role in the regulation of gene expression [1]. Specifically, methylation of regulatory regions can prevent binding of specific transcription factors or repress transcription by recruiting chromatin remodeling proteins [2]. In mammalian organisms, $\sim$80% of CpG dinucleotides are methylated. Notable exceptions are CpG islands (CGI), regions of $\sim$300–3000 base pairs that are rich in CpG dinucleotides [3]. Researchers have documented the existence of hypermethylated CGIs in a battery of cancers. This ‘CpG island methylator phenotype (CIMP)’ can favor cancer progression by repressing tumor suppressor genes through promoter methylation [4, 5].

Initially described in colorectal cancer [6], CIMP was later documented in bladder [7], breast [8], cervical [9, 10], endometrial [11], esophageal [12], gastric [13, 14], head and neck [15, 16], hepatocellular [17], lung [18–20], pancreatic [21], prostate [22] and thyroid cancer [23, 24], adrenocortical [25] and renal cell carcinoma [26], duodenal adenocarcinomas [27], glioma [28, 29], leukemia [30, 31], melanoma [32], neuroblastomas [33] and thymoma [34]. In these studies, the presence of CIMP often resulted in tumor suppressor gene promoter methylation, was linked to clinicopathological patterns such as stage, and was often associated with better or worse prognosis [35], supporting the potential use of CIMP status as a clinical marker to predict cancer progression but highlighting differences in downstream molecular processes across cancer types.

But although the topic remains studied and discussed in recent years [36–40], surprisingly no universal definition of CIMP has emerged. This absence may be because the phenotype was seemingly cancer-type-specific [35]. Indeed, when investigators used Weisenberg *et al*.’s colorectal cancer gene panel [41] to study CIMP in their cancers of interest, the researchers often found no clear evidence of gene-CIMP linkage.

The need for a universally accepted definition of CIMP has heightened with the emergence of tautological definitions of CIMP tumors (those with high-methylation profiles) and contradictory findings about the effects of CIMP. The confusion may be linked to the current lack of understanding of the underlying molecular drivers of CIMP, and therefore investigators have conducted molecular studies and made significant inroads. For example, researchers found causation between mutations in *IDH1*, *IDH2* and *TET2*—which negatively affect DNA hydroxymethylation rates—and CIMP in leukemia [42], gliomas [29] and several other cancer types [35]. Such results open the possibility of a clearer definition of CIMP, cancer by cancer.

Admittedly, other researchers have attempted to identify a common CIMP etiology through a pan-cancer approach [43–47]. However, none have corrected for biases in age or tumor purity, which can distort DNA methylation signals. Furthermore, most studies compared only hypermethylated CpG probes to normal tissue [43, 44, 46, 47], and some involved merely a small number (5–15) of cancers and/or deployed methodologies (e.g. *k*-means clustering) that were not specifically designed for clusters with significant variation in size, as would be expected for cancers with a low prevalence of particular causal mutations [43, 46].

More specifically, Karpinski *et al*. [44] implicated the existence of CIMP in all 23 cancer types investigated; however, the difference in average methylation between the high-methylation and low-methylation groups could be as low as 0.01. Similarly, Moarii [45] reported CIMP in all five cancer types studied. In addition, the elegant work of Yang *et al*. [46] and Saghafinia *et al*. [47] tracked differentially methylated positions and global methylation dysregulation overall but did not study CIMP specifically.

Given this backdrop, we aimed to: (i) define CIMP reliably through a pan-cancer approach, analyzing methylation values in the most variable probes of CGIs and removing the effects of age, gender and tumor purity; (ii) identify candidate driver events for CIMP through gene mutation and expression analyses and (iii) analyze CIMP’s effect on patient survival unraveling its potential downstream effects. The results show the variety of CIMP manifestations and diversity of potential causes in different cancer types. The work also demonstrates the value of profiling DNA methylation in cancers to stratify patient risk and customize treatment.

## Materials and methods

### Datasets

We studied The Cancer Genome Atlas (TCGA) dataset (https://www.cancer.gov/about-nci/organization/ccg/research/structural-genomics/tcga), selecting cancer types that offered at least 80 samples with associated methylation information in the Genomic Data Commons (GDC) data portal (https://portal.gdc.cancer.gov/). Our study encompassed 26 cancer types (see List of Acronyms). Normal samples were extracted from the TCGA dataset (when available). For low grade glioma (LGG) and mesothelioma (MESO), we used glioblastoma multiforme (GBM) and lung adenocarcinoma (LUAD) normal samples, respectively. For adrenocortical carcinoma (ACC) and acute myeloid leukemia (LAML), no healthy tissue samples were available in TCGA. Thus, we downloaded normal tissue methylation expression arrays from the Gene Expression Omnibus (GEO) series GSE77871 and GSE32149 (list of GEO accession numbers in Supplemental Appendix).

We used preprocessed molecular data characterizing DNA methylation, gene expression and genomic variants (Supplemental Methods).

### Preprocessing DNA methylation data

**Figure 1:**
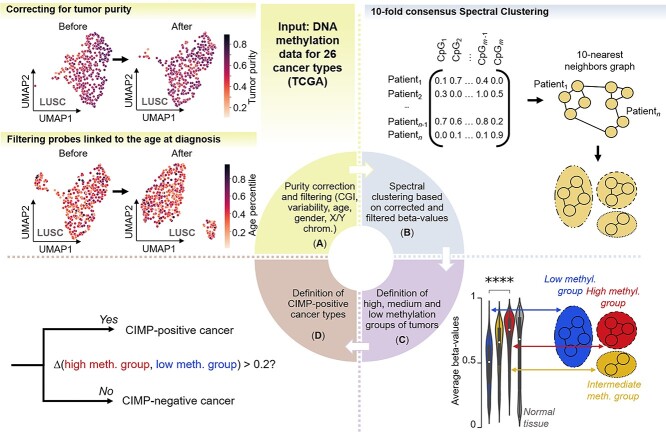
Pipeline for CIMP classification in a pan-cancer context. (**A**) Using 26 cancer types from the TCGA dataset, we first selected probes located in shelves, shores and CpG Islands only, filtered out nonvariable probes, corrected for purity effects and filtered out age and gender-related probes. Example of the effect of purity correction and age filtering is given for lung squamous cell carcinoma; tumor samples with unknown purity values were not included in the purity correction plot. (**B**) We used the beta-value vectorial representation of patients to construct a 10-nearest neighbor graph and performed 10-fold consensus spectral clustering; we obtained two to three clusters for each cancer type. (**C**) We defined low, intermediate and high-methylation groups based on the average beta-values in each cluster of patient samples. (**D**) We compared the average methylation values over significantly differentially methylated probes between the high-methylation and low-methylation group to determine which cancer types presented CIMP.

We sought to avoid any artificial grouping of tumor samples linked to gender, age or high proportion of non-malignant cells with their specific methylation signal while preserving the potential link between these variables and a specific cancer subtype [48] (Figure 1). We chose to employ extensive data processing after reviewing several studies [64, 65] that demonstrated the ability of heterogeneous tissue cell composition to bias downstream analysis, increase within-group variation and mask valid signals. In addition, numerous studies [66–69] have highlighted the effect of age and gender on methylation values in a nontissue specific manner, which can also confound analysis results.

First, we removed non-CGI probes, restricting the analysis to CGIs, CGIs’ ‘shores’ (genomic regions up to 2 kilobases from CGIs) and ‘shelves’ (2 to 4 kilobases from CGIs) [49]. We removed probes for which there was no information in at least one patient per cancer cohort [50]. As a first step to avoid gender biases, we also removed CpG dinucleotides located on the X and Y chromosomes.

Next, we removed potential noise in the data [43, 45] by selecting the most variable probes. We computed the distribution of standard deviation (SD) for each probe and cancer type and then performed *k*-means clustering in the SD space (with *k* = 2). We further removed the probes belonging to the cluster with the lowest centroid, corresponding to the set of nonvariable probes (Supplemental Figure 1).

We then corrected for tumor purity, preserving the potential signal linked to cancer subtypes. Before the correction, we did observe a strong bias toward tumor purity in several cancer types, including kidney renal clear cell carcinoma (KIRC) and lung squamous cell carcinoma (LUSC) (Supplemental Figure 2). Thus, we deconvolved the tumor DNA methylation values into signals from cancerous and nonmalignant cells and used only the former. We used the models developed in debCAM [51] and a subtype-specific approximation of individual methylation levels, as described in Chen *et al*. [52] (Supplemental Methods).

Finally, we removed potential pan-cancer age- and gender-related CpG positions, retaining only those linked to the patient age or gender in a particular cancer subtype. Specifically, we first removed age-related CpG positions detected by Slieker *et al*. [53]. Then, for each cancer type, we computed the correlation between age (resp. gender) and CpG probes and recorded the CpG probes associated with age (resp. gender) [false detection rate (FDR) *q* < 0.05] in at least two cancer types. We then removed from the remaining probes all probes identified as associated with age (resp. gender) in any two cancer types. We reasoned that subtype-related probes we were interested to preserve should not be present in other cancer types (Supplemental Figure 3).

### Clustering DNA methylation data

To detect groups of tumor samples with similar DNA methylation profiles, we applied unsupervised clustering on age-filtered and purity-corrected, highly variable DNA-methylation beta-values. We used spectral clustering [54] with the 10-nearest neighbor affinity matrix. We implemented 10-fold consensus clustering to avoid randomness due to initialization (Figure 1). Of note, we compared spectral clustering against several clustering algorithms. We chose spectral clustering because it best separated our data, according to the average silhouette score (Supplemental Table 1).

To characterize the group of tumors with similar DNA methylation profiles (obtained with spectral clustering), we first compared the distributions of beta-values between the clusters using a Kruskal–Wallis test with Bonferroni correction. We then computed for each cluster the average methylation value per CpG probe over all significant positions. We refer to the cluster with the smallest average methylation value mean over all CpG positions as the low-methylation group. We refer to the group with the highest value as the high-methylation group. We have determined in some instances an intermediate-methylation group. For each cancer type, we chose at least two distinct DNA methylation groups, based on the separability of the low- and high-methylation clusters (Supplemental Methods).

To measure the uncertainty linked to the clustering of a patient within a methylation group, we computed the sample silhouette coefficient (SSC) with Euclidean distance. Generally, samples with negative values of SSC could be attributed to two different methylation clusters with a similar probability. We refer to patients with SSC greater than 0 as ‘high confidence’ (HC) patients.

To detect CIMP subsets, we compared DNA methylation values in the high- and low-methylation groups. We deemed a cancer type CIMP-positive if the difference in the average beta-values between the high and low groups was greater than 0.20 and the distributions were significantly different (Figure 1).

Finally, to elucidate potentially artificial grouping linked to a specific clinical or molecular subtype for each cancer, we computed the correlation between cluster membership and available clinical information (Kruskal–Wallis for continuous, Chi-square for categorical, Bonferroni-corrected *P*-values). We identified one case, pheochromocytoma and paraganglioma (PCPG), in which two DNA methylation groups clearly corresponded to cancer subtypes. Thus, we excluded the paraganglioma subtype (consisting of only 39 patients) and included only pheochromocytoma (PC) (*n* = 152) for further analysis.

### Mutation analysis

To detect possible genomic drivers of CIMP, we identified relevant mutations in the methylation groups using only HC patients. We first computed the percentage of patients within a group that carried a mutation within a list of genes associated with DNA and histone methylation and demethylation (listed in Supplemental Appendix).

For all cancer types and genes, we computed an associated *P*-value with Fisher’s exact test, corrected by the Benjamini–Hochberg method. We reported only significant mutations (*P* < 0.05) with a mutation frequency difference between the low- and high-methylation groups greater than 10%. We also indicated mutations that did not pass the FDR 0.1 threshold as nonsignificant (NS). We compared microsatellite instability (MSI) status in each group for colon adenocarcinoma (COAD) and uterine carcinoma (UCEC), using the TCGA consortium calling [55]. Deploying Fisher’s exact test, we calculated the enrichment of MSI high, as compared with microsatellite stable, in the high- versus low-methylation groups.

### Random forests for mutation discovery

We trained Random Forest classifiers on HC patients to identify putative CIMP-driving mutations per cancer type in genes other than those associated with methylation or demethylation. We sought to capture nonlinear and nonadditive effects of genomic mutations. We used the full mutational information to predict group membership for each cancer type and analyzed the selected features.

### CIMP score and mutation correlation

Considering the gradient-like nature of certain groups [e.g. in adrenocortical carcinoma (ACC)] as opposed to a more subtype-like nature (e.g. in LGG), we introduced a continuous CIMP score, consisting of the average beta-value over all significantly differentially methylated probes between the high- and low-methylation groups for each patient. Patients were then ranked according to their CIMP score, and the point-biserial correlation between the CIMP score and their gene mutations was computed.

### CIMP and patients’ clinical outcome

To assess whether DNA methylation groups are associated with distinct clinical outcomes, we computed the Kaplan–Meier estimator for each cancer methylation group. We also performed a log-rank test to compare survival between the groups, using the Benjamini–Hochberg correction [56] and only HC patients for modeling. To ascertain whether DNA methylation groups provide added value in addition to clinical variables in patient risk stratification, we trained a Cox regression model [57] correcting for age, gender and stage, when available or relevant. No stage information was provided for GBM, LGG, PCPG or sarcoma (SARC), and no gender correction was performed for cervical squamous cell carcinoma and endocervical adenocarcinoma (CESC). Both investigations were performed using the Python lifelines library [58] and used the survival information derived from the cleaned pan-cancer initiative from Liu *et al*. [59].

To investigate how CIMP could be effectively assessed in the clinic, we searched for a set of up to five probes that could predict CIMP status with near perfect accuracy. We trained a logistic regression on each significantly differentially methylated probe (90%/10% training/test split, balanced class weights, scoring done with 5-fold stratified cross validation and adjusted balanced accuracy as described previously to predict the CIMP status). We used Sequential Forward Selection [60] to select *n* optimal probes for classification (*n* = 5).

### Analysis of downstream transcriptional changes and tumor microenvironment

To investigate how the CIMP status might influence biological processes in cancer cells, we first selected genes both differentially expressed between the high- and low-methylation groups and associated with hypermethylated probes in the high-methylation group; we required more than a 10% difference in beta-values between groups. We refer to this set of genes as potential CIMP downstream targets. We then used the method developed for the Database for Annotation, Visualization and Integrated Discovery (DAVID) [61] (modified version of Fisher’s exact test) to find enriched gene sets in the Gene Ontology Biological Processes [62] and KEGG pathways [63], using the updated version of the databases (v7.4) retrieved from the Broad Institute website (https://www.gsea-msigdb.org/gsea/index.jsp).

Independently, we attempted to identify cellular characteristics of the tumor microenvironment (TME) that were potentially associated with CIMP status. Using Fisher’s exact test, we first computed significant enrichments in the immune subtypes described in Thorsson *et al*. [64] [wound healing, interferon gamma (IFN-γ) dominant, inflammatory, lymphocyte depleted, immunologically quiet, transforming growth factor beta (TGF-β) dominant].

Next, we computed the Spearman correlation coefficient *R* between the CIMP score and immune signatures scores and characteristics. We also computed *R* between the CIMP score and precomputed estimates from CIBERSORT [65] and xCell [66] computational methods to characterize cell composition of complex tissues through gene expression profiles. We obtained both from Thorsson *et al*. [64]. We investigated associations with leukocyte fraction of the TME, proliferation and three immune signatures: wound healing [67], macrophage regulation [68] and lymphocyte infiltration [69].

### Statistical tests used in the analysis

For all correlation analyses (Pearson, Spearman and point-biserial correlations), we used a t-test to compute associated *P*-values, corrected by Benjamini–Hochberg FDR to obtain *q*-values. The level of significance was *q* < 0.05 for age- and gender-related probe filtering and immune composition, and *q* < 0.1 for mutation correlation with CIMP score.

To obtain probes significantly differentially methylated between methylation groups and compare the distributions of average beta-values between these groups, we used the Kruskal–Wallis test to calculate *P*-values, corrected with Bonferroni correction.

To study significant enrichment in clinical values in our clusters, we used the Kruskal–Wallis test for continuous and Chi-square test for categorical ones, with the Bonferroni correction of *P*-values.

To study associations between cluster membership and mutations or immune subtypes, we used the Fisher’s exact test and used Benjamini–Hochberg FDR correction to obtain *q*-values.

For survival analyses, we used the log-rank test corrected with Benjamini–Hochberg FDR for univariate analyses, and Wald test for the Cox regression, uncorrected.

For pathway enrichment analysis, we used the EASE score, a modified version of Fisher’s exact test developed for the DAVID [61] tool, corrected with Benjamini–Hochberg FDR.

## Results

### All 26 analyzed cancer types demonstrated dysregulation of DNA methylation; 19 showed a global CGI hypermethylation pattern

Based on the literature, we defined CIMP as the existence of a subset of patients displaying significantly higher CGI DNA methylation compared with another subset, [8, 10, 13, 14]. Using informative CpG probes, we then characterized CIMP prevalence in 26 cancer types, removing potential biases linked to tumor purity, age or gender.

To account for biases linked to age, gender or tumor purity that might confound the analysis, we used only the most variable probes, corrected for tumor purity [51, 52], and filtered out all potentially age- and gender-related probes (Methods). Notably, we did not screen for probes that were differentially methylated as opposed to normal tissue, but rather a posteriori compared DNA methylation levels between the CIMP tumor subset and nonmalignant controls.

**Figure 2:**
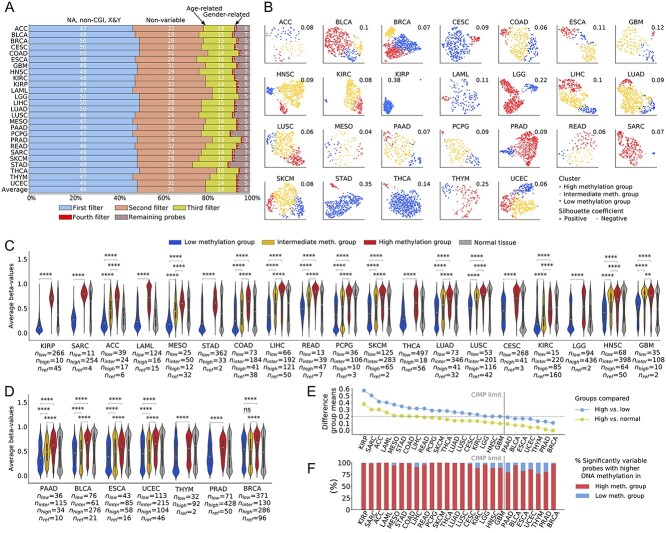
Discovery of cancer types presenting characteristics of CIMP. (**A**) Percentage of probes removed by each filter. The first filter removed NA probes, probes not located on CGI (specifically CGIs, shores or shelves) and probes located on the X and Y chromosomes. The second filter removed nonvariable probes. The third filter removed potentially age-related probes. (**B**) UMAP representation of the results of spectral clustering based on purity-corrected DNA methylation values of filtered CpG probes. The groups are indexed according to their average beta-value over all significantly differentially methylated probes. Average silhouette coefficients are indicated at the top right of each cancer type. (**C**) Distribution of average beta-values over significantly differentially methylated probes for 19 CIMP-positive cancer types. (**D**) Distribution of average beta-values over significantly differentially methylated probes for seven CIMP-negative cancer types. Cancer types are ranked according to the difference in average beta-values between the high-methylation and low-methylation group. Significance is computed with a Kruskal–Wallis test. Distribution of beta-values for normal reference tissue is displayed in gray next to distribution of cancerous samples. Sizes of groups are indicated as *n_low_*, *n_inter_* and *n_high_* for low, intermediate and high-methylation, respectively, with n_ref_ being the number of the normal reference samples. The white circle indicates the median and the inner box plot indicates the lower and upper quartile. Significance is reported for Bonferroni-corrected *P*. NS: *P* > 0.05; ^*^: 0.01 ≤ *P* < 0.05; ^*^^*^: 0.001 ≤ *P* < 0.01; ^*^^*^^*^: 0.0001 ≤ *P* < 0.001; ^*^^*^^*^^*^: *P* < 0.0001. (**E**) Percentage of probes hyper- or hypomethylated in the high- versus low-methylation group, computed on the set of probes significantly differentially methylated between groups. (**F**) Difference in mean beta-value between the high and low-methylation groups and high-methylation group and normal tissue, computed using all significantly differentially methylated probes. The cut-off value for CIMP-positiveness (0.2) is indicated by a horizontal line. BLCA: bladder urothelial carcinoma; BRCA: breast invasive carcinoma PAAD: pancreatic adenocarcinoma; READ: rectum adenocarcinoma; THYM: thymoma.

After preprocessing, the average number of informative methylation sites per cancer type was 28 218 [IQR (24 473–31 734)] (Figures 1A and 2A, Supplemental Table 2). Any tumor purity-linked gradient that was present disappeared after deconvolution (Figure 1B, Supplemental Figure 2).

Using spectral clustering, we grouped patients according to their DNA methylation profiles (Methods) and utilized this profiling to characterize cancer types. We identified the optimal number of clusters that would maximize the separability between low- and high-methylation clusters (Supplemental Methods, Supplemental Table 3): 2 clusters for 10 of the cancer types and 3 clusters for the remaining 16 (Figure 2B). For several cancers, clustering structure was apparent in the two-dimensional Uniform Manifold Approximation and Projection (UMAP) representation (e.g. GBM), suggesting distinct subtypes within the cancer type. For the remainder, the boundary between clusters resembled a gradient (e.g. ACC).

We computed the sample silhouette score (SSC) as a measure of uncertainty of cluster membership (Figure 2B, Supplemental Figure 4, and Supplemental Table 2). We classified tumors by their methylation status i.e. high, intermediate and low methylation (Methods). Overall, the mean value of differentially methylated CpGs was 11 914 [IQR (5211–19 213)] (Supplemental Table 2). Because the intersection of the CpG probes selected for all cancer types was empty, we found that a unique panel of CpG probes cannot be constructed to identify CIMP in a pan-cancer manner.

To identify patient clusters potentially linked to underlying clinical features (e.g. cancer subtypes), we performed correlation analysis between cluster membership and clinical features. We discovered several significant relationships (Supplemental Table 4), most of which were linked to patients’ age at diagnosis, survival status and stage.

We classified cancer types into CIMP-positive and CIMP-negative, based on the differences in cluster-wise average values of DNA methylation (Methods). Although CIMP had previously been reported in all 26 studied cancer types, our analysis showed only 19 cancer types as CIMP-positive (Figure 2C–E and Supplemental Table 3). Of note, we observed two types of CIMP-positive DNA hypermethylation: that targeting predominantly CGIs and that targeting shelves and shores, as well CGIs (Supplemental Figure 5).

In all cancers, the average beta-value distributions were significantly different between groups (as measured by Kruskal–Wallis adjusted with Benjamini–Hochberg correction). Across cancers, only 6% of probes [IQR (1–11%)] were hypomethylated in the high-methylation group as compared with the low-methylation group (Figure 2E). We concluded that the vast majority of probes in the high-methylation group become hypermethylated individually, as well as the group displaying a higher degree of methylation overall.

We did not choose our probes a priori to be more methylated than in the normal reference tissue. However, we observed that the probes selected for the analysis did present a consistently positive difference in mean beta-values between the high-methylation group versus normal tissue (Figure 2F).

Overall, we found apparent epigenetic dysregulation in all 26 cancer types studied, in the form of *both* hypo- and hypermethylation as compared with normal tissue, consistent with previous reports [47].

### Among DNA and histone methylation and demethylation genes, only isocitrate dehydrogenases *IDH1*/*2* and histone methyltransferase *SETD2* mutations are reproducible drivers of CIMP

Based on reports of extensive interactivity between histone and DNA methylation in relation to cancer [70, 71], we analyzed differences in mutations within genes associated with DNA and histone methylation or demethylation. Our aim was to identify potential drivers of DNA hypermethylation in CIMP-positive cancers.

**Figure 3:**
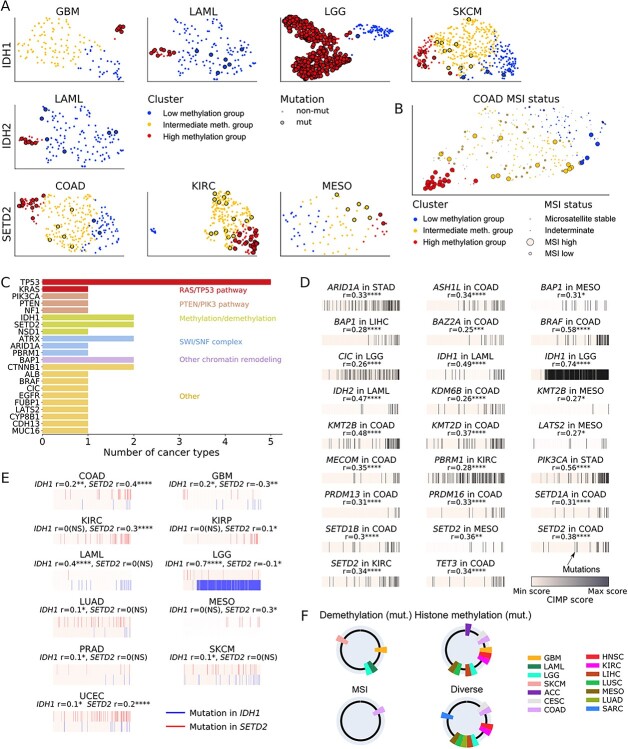
Discovery of possible drivers of CIMP. (**A**) UMAP representation of patients with mutations in *IDH1/2* or *SETD2* genes. (**B**) MSI status of COAD. MSI annotations are taken from the TCGA consortium calling, using Cortes-Ciriano [55] Supplementary Tables, indeterminate status is colored in gray. (**C**) Genes selected in the Random Forest analysis as potential drivers for either low, intermediate or high-methylation groups and their associated pathways. We trained Random Forests for 10 CIMP-presenting cancer types. The number of cancer types for which the gene was selected is indicated. (**D**) Mutations significantly correlated with CIMP score and (**E**) mutations in *IDH1* and *SETD2* significantly correlated with CIMP score. Patients are ranked for each cancer type according to their CIMP score. Patients presenting a mutation in the gene of interest are indicated by a black bar, in *IDH1* by a blue bar and in *SETD2* by a red bar. Point biserial correlation coefficient *r* is indicated. For (**D**), only mutations with a correlation coefficient (*r*) over 0.25 are shown; the full mutation panel is depicted in Supplemental Figure 9. Significance is reported for FDR Benjamini–Hochberg-corrected *q*-value for (**D**) and (**E**). NS: *q* > 0.1; ^*^: 0.01 ≤ *q* < 0.1; ^*^^*^: 0.001 ≤ *q* < 0.01; ^*^^*^^*^: 0.0001 ≤ *q* < 0.001; ^*^^*^^*^^*^: *q* < 0.0001. (**F**) Potential etiologies of CIMP in 19 CIMP-positive cancer types. Cancer types are represented as a portion of the circle with an associated color. The four circles represent the four candidate etiologies for CIMP in these cancer types: mutations in the DNA demethylation associated genes, mutations in the histone methylation associated genes, MSI and diverse or unelucidated mechanisms.

We identified 10 cancer types that exhibited differences in mutational frequency of greater than 10% between the high- and low-methylation groups (Table 1). Cancer types that displayed the largest differences between groups were LGG (*IDH1* 1% in the low- versus 95% in the high-methylation group), GBM (*IDH1* 0% versus 88%), LAML (*IDH1* 6% versus 56%; *IDH2* 3% versus 44%) and KIRC (*SETD2* 0% versus 36%) (Figure 3A).

**Table 1:** Putative drivers of CIMP among mutations and gene expression changes for 19 CIMP-positive cancer types

**Cancer**	**(De)Methylation mutation**	**Diff. GEX**	**Non-methylation mutation**
**ACC**	KMT2A (0%/14%)^*^	PRDM13 (0.2)^*^	MUC16 (5%/12%) (NS)
**CESC**	KMT2D (16%/5%)^*^	PRDM13 (0.3)^*^, PRDM16 (1.5)^*^, TDGF1 (2.8)^*^	-
**COAD**	ASH1L (0%/33%)^*^, EHMT1 (0%/13%)^*^, EHMT2 (2%/13%)^*^, KMT2A (8%/23%)^*^, KMT2B (5%/67%)^*^, KMT2C (12%/31%)^*^, KMT2D (8%/56%)^*^, MECOM (0%/23%)^*^, NSD1 (0%/15%)^*^, PRDM1 (0%/13%)^*^, PRDM2 (2%/15%)^*^, PRDM9 (3%/23%)^*^, PRDM10 (2%/18%)^*^, PRDM13 (0%/13%)^*^, PRDM15 (2%/15%)^*^, PRDM16 (2%/26%)^*^, SETD1A (0%/23%)^*^, SETD1B (2%/31%)^*^, SETD2 (3%/36%)^*^, SETDB1 (2%/13%)^*^, KDM2B (7%/28%)^*^, KDM3B (2%/13%)^*^, KDM4A (0%/18%)^*^, KDM4B (0%/15%)^*^, KDM5A (2%/13%)^*^, KDM5B (2%/15%)^*^, KDM6A (2%/13%)^*^, KDM6B (3%/26%)^*^, PHF2 (2%/13%)^*^, TET1 (2%/18%)^*^, TET2 (2%/13%)^*^, TET3 (3%/33%)^*^, MBD1 (2%/13%)^*^, CTCF (0%/10%)^*^, BAZ2A (0%/15%)^*^, DNMT1 (0%/15%), UHRF1BP1L (2%/18%%)^*^	PRDM13 (0.2)^*^, PRDM8 (1.3)^*^, TDGF1 (0.7)^*^	KRAS (23%/20%) (NS) BRAF (0%/75%), TP53 (81%/40%)
**GBM**	**IDH1 (0%/88%), ATRX (16%/62%)**	-	**TP53 (44%/100%)**
**HNSC**	NSD1 (72%/4%), PRDM9 (17%/0%)^*^	PRDM13 (2.2)^*^, PRDM8 (0.8)^*^, CTCFL (3.6)^*^	-
**KIRC**	**SETD2 (0%/36%)**	TDGF1 (2.4)^*^	PBRM1 (0%/58%)
**KIRP**	-	-	-
**LAML**	**IDH1 (6%/56%), IDH2 (3%/44%),**	-	-
**LGG**	**IDH1 (1%/95%), ATRX (7%/45%)**	PRDM13 (3.2)^*^, SMYD1 (1.3)^*^, TET1 (0.8)^*^	EGFR (37%/0%), CIC (1%/26%), FUBP1 (0%/11%), **TP53 (15%/56%)**, NF1 (20%/3%), **PTEN (22%/1%)**
**LIHC**	-	PRDM6 (1.3)^*^, PRDM16 (1.7)^*^, PRDM9 (0.4)^*^	CTNNB1 (27%/8%)^*^, **BAP1 (0%/16%)**, ALB (14%/4%)^*^, TP53 (41%/18%),
**LUAD**	-	CTCFL (2.4)^*^	
**LUSC**	NSD1 (55%/3%)^*^, SETD1A (14%/3%)^*^ (NS)	CTCFL (3.1)^*^	CYP8B1 (10%/0%)
**MESO**	KMT2B (0%/18%)^*^ (NS), SETD2 (0%/18%)^*^ (NS)	-	**BAP1 (0%/36%)**, LATS2 (4%/45%)^*^
**PCPG**	-	-	
**READ**	-	-	CDH13 (27%/0%)
**SARC**		CTCFL (4.4)^*^	-
**SKCM**	**IDH1 (3%/14%) (NS)**	-	-
**STAD**	-	-	**PIK3CA (11%/75%), TP53 (53%/12%), ARID1A (22%/50%), CTNNB1 (5%/19%),**
**THCA**	-	-	**-**

The putative mutations were extracted from the mutation analysis with the Random Forest method. Differential gene expression (Diff. GEX) was computed through DESeq2 between high and low-methylation groups for genes involved in DNA and histone methylation. Mutations are indicated as low-methylation group %/high-methylation group %, gene expression is indicated as fold change (FC) between high- and low-methylation groups. (FC > 1 corresponds to overexpression in the high-methylation group). Only significant mutations (Fisher exact test *P* < 0.05) with a difference > 10% between the low- and high-methylation groups were reported. Mutations that did not pass the 0.1 threshold on *q*-value are indicated by NS. Cancer types for which the indicated mutations had not yet been described in relationship with CIMP are indicated by an asterisk (^*^). Candidate driver mutations or differential gene expression shared among at least two cancer types are indicated in bold.

In the case of COAD, we discovered that MSI was significantly enriched in the high-methylation group, consistent with previous reports [41, 47] (Figure 3B, *P* = 4.1 × 10^−9^). To account for mutational burden in tumors with MSI, we corrected mutation frequency with overall mutation rate and computed an empirical *P*-value associated with enrichment (Supplemental Methods, Supplemental Table 5). We discovered that 38% of COAD-enriched genes were mutated more than by chance, including *KMT2B* (5% low- versus 65% high-methylation group). Of note, we discovered that MSI was significantly enriched in the high-methylation group of UCEC as well (Supplemental Figure 6, *P* = 1.8 × 10^−9^).

We repeatedly found mutations in the *NSD1* gene in the low-methylation group of head–neck squamous cell carcinomas (HNSC) (72% versus 4%) and LUSC (55% versus 2%) suggesting a common mechanism of hypomethylation (Supplemental Figure 7).

### Mutations in genes not directly involved in methylation are associated with CIMP in several cancer types

To discover potential mutational drivers in genes other than those involved in DNA and histone methylation or demethylation, we trained Random Forest classifiers on the full mutation data. The rationale was that Random Forest features (i.e. mutations) typically used by the algorithm for CIMP status prediction might be biologically relevant in CIMP etiology. We identified 10 cancer types for which the Random Forest detected potential driver mutations, performing better than a random classifier to predict samples with CIMP (Supplemental Methods, Supplemental Figure 8).

The results confirmed known associations (Figure 3C), such as cancer-related genes involved in the Ras/TP53 pathway [e.g. *TP53* (5/10 cancer types) and *KRAS* (1/10)]; genes involved in chromatin remodeling by the SWItch/Sucrose Non-Fermentable (SWI/SNF) complex [e.g. *ATRX* (2/10) and *ARID1A, PBRM1* (1/10 each)] and genes previously associated with CIMP [e.g. *BAP1* (2/10)]. We confirmed the association with CIMP of genes primarily involved in methylation or demethylation, such as *IDH1* (2/10) and *SETD2* (2/10). Of note, we found several mutations in genes identified within the low-methylation group to be useful for classification, including *NSD1* (1/10 cancers).

### Mutations associated with CIMP are correlated with a continuous CIMP score

Considering the gradient-like nature of clustering in some cancer types (Figure 2B), we introduced a continuous CIMP score and computed the point biserial correlation between the score and gene mutations. Comparing groups, we discovered that most significantly enriched mutations were correlated with a cancer’s CIMP score, further confirming the potential link between these mutations and hypermethylation events. Based on their UMAP representation, groups that exhibited a gradient-like structure also displayed a gradual enrichment of identified mutations, whereas groups that exhibited a subtype-like structure displayed a more abrupt mutational enrichment (Figure 3D and Supplemental Figure 9). Of note, we found that *KRAS* mutations were enriched in the intermediate COAD group, consistent with previous reports [72] (Supplemental Figure 9).

Finally, as we found that only *IDH1* and *SETD2* mutations were identified as potential genomic drivers of CIMP across more than two cancer types, we investigated whether there might be small groups of patients presenting mutations in *IDH1* or *SETD2* correlated with hypermethylation that would remain undetected by our method. Indeed, the cluster size required by our approach for DNA methylation groups may be too large to detect signals coming from a very small portion of the samples. We thus searched for patients presenting a mutation in *IDH1* or *SETD2* and then computed the point-biserial correlation between the mutation status and the CIMP score. Other than the seven previously reported cancer types presenting significant mutations in either *IDH1* or *SETD2* in the high-methylation group, we found four additional cancer types with small groups of patients whose mutational status significantly correlated with the increased CIMP score (Figure 3E, Supplemental Table 6). These observations indicated that the *IDH1* and *SETD2* mutations might be potential genomic driver events of CGI hypermethylation in a large variety of cancer types. The percentage of the cancer samples affected by the putative driver mutations was 6% for kidney renal papillary cell carcinoma (KIRP), 1% for LUAD and 1% for prostate cancer (PRAD) (Supplemental Table 6). Of note, although the presence of the *IDH1* and *SETD2* mutations was significantly correlated with hypermethylation in UCEC, this effect was hard to deconvolve from the MSI status (Supplemental Figure 6).

### *BORIS*/*CTCFL*, recently linked to changes in DNA methylation, is differentially expressed between low- and high-methylation groups in four cancer types

We further hypothesized that aberrations in the transcriptional levels of genes related to DNA or histone methylation can potentially drive CIMP in certain cancers. We used DESeq2 to investigate differential gene expression between high- and low-methylation groups in the 19 identified CIMP-positive cancers.

Our results showed that the transcription of the ‘modulator brother of regulator of imprinted sites’ (BORIS), also known as CCCTC binding factor-like (CTCFL), was upregulated in the high-methylation group of four types of cancers (3.6-fold change for HNSC, 2.4 for LUAD, 3.1 for LUSC and 4.4 for SARC). We hypothesized that mutated BORIS/CTCFL might displace the highly conserved zinc finger protein CTCF that protects CGIs from methylation in healthy cells, thereby promoting aberrant hypermethylation [2].

### Mutations and gene expression changes between DNA methylation groups suggest four main potential etiologies for DNA hypermethylation

Combining the analyses of mutations and gene expression changes in different DNA methylation groups, we arrived at four types of etiologies that might underlie a CIMP presentation in a cancer type (Table 1 and Figure 3F). The first, represented by COAD, involved a high-methylation group coincident with tumors exhibiting MSI [73]. This group presented a high number of mutations in genes involved in DNA methylation, alongside with genes responsible for methylation of histone residues H3K4, H3K9 and H3K36, such as the *KMT2* gene family and *SETD2*. Of note, *SETD2* was previously linked to CIMP in KIRC [74] but not COAD.

The second category, represented by GBM, LGG and LAML, showed CIMP drivers to be mutations in the DNA demethylation genes *IDH1/2*, as previously reported [29–31]. To a lesser extent, we found additional *IDH1* mutations in high-methylated skin cutaneous melanomas (SKCM), in accord with previous studies [75]. Of note, we found that small groups (1%) of LUAD and PRAD presenting *IDH1* mutations had a higher methylation level, consistent with previous reports for PRAD [76]. *IDH1* mutations in LUAD have previously been reported as rare events and potential drivers of subclonal evolution [77] but not of CIMP.

The third category was based on mutations in genes involved in histone methylation or demethylation. The main driver of CIMP appeared to be the SET domain-containing family, whereby the loss of function of *SETD2* is associated with hypermethylation events and can lead to ectopic H3K36me3 [78]. We observed a significant increase in *SETD2* gene mutations in the high-methylation group of COAD (2% low- versus 36% high-methylation group), KIRC (0% versus 37%) and MESO (0% versus 18%). We also found that *SETD2* mutations were significantly correlated with a higher methylation level in KIRP. Supporting this finding, studies have reported *SETD2* mutations to be characteristic of a certain group of renal cancers associated with CIMP [74]. Interestingly, our results showed a significant increase in *NSD1* mutations in the low-methylation groups of several cancer types, in accord with previous results [79, 80]. NSD1 is also a SET domain-containing protein involved in methylation of H3K36 and known to recruit DNMT3A/B to gene bodies [70].

The fourth category involved four cancer types in which the CIMP etiology was discernible, based on our mutational analysis and a literature search. For CESC, DNA hypermethylation may be caused by the HPV E7 viral protein [81]. In MESO and liver hepatocellular carcinoma (LIHC), *BAP1* mutations were enriched in the high-methylation group, in accord with previous studies [74]. We observed *BORIS/CTCFL* overexpression in the high-methylation groups of HNSC, LUSC, LUAD and SARC. Finally, mutations in *BRAF*, *KRAS*, *PBRM1* and *PTEN* were found in the hypermethylated groups of COAD, KIRC and LGG, consistent with previous analyses [43].

### CIMP is a prognostic factor in numerous cancer types and can be cost-effectively assessed in the clinic

**Figure 4:**
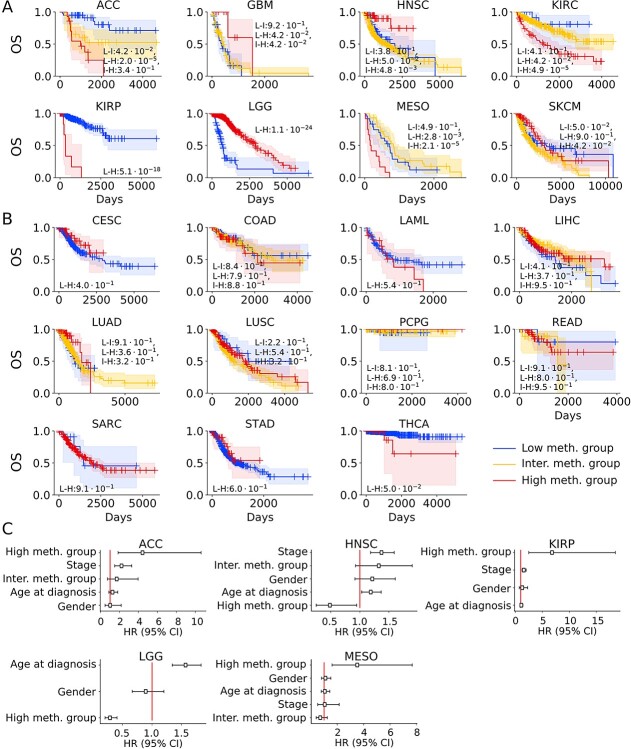
CIMP as a prognostic factor for patients’ clinical outcome. (**A**) Kaplan–Meier representation of univariate overall survival (OS) analysis for eight cancer types with significant differences in OS between DNA methylation groups. (**B**) Kaplan–Meier representation for 11 cancer types without significant differences in OS between DNA methylation groups; 95% confidence interval (CI) is represented by a colored area around the Kaplan–Meier curve. The associated log-rank test *P-*value is indicated as low- versus high-methylation group (L-H), and when relevant, low versus intermediate (L-I) and intermediate versus high-methylation groups (I-H). (**C**) Cox regression model representation of hazards for significant associations. The hazard ratios (HR, with 95% CI) associated with each variable for all significant cancer types are represented. High-methylation group (resp. inter. meth. group) quantifies the hazard ratio associated with belonging to the high-methylation (resp. intermediate-methylation) as compared with the low-methylation group.

To investigate the role of CIMP as a survival predictor and independent prognostic marker, we performed both univariate and multivariate analyses, using log-rank tests and Cox proportional hazard models. We identified eight cancer types with significantly different survival times across DNA methylation groups: ACC, HNSC, KIRC, KIRP, GBB, MESO, SKCM and LGG (Figure 4A and B and Supplemental Table 7). The link between patient survival and CIMP for all but HNSC had been previously reported in literature [25, 26, 28, 29, 74, 82].

Reports of the contribution of the CIMP status to survival in SKCM have been mixed [83–86]. Our analysis suggested that the cancer type violated the Cox proportional hazard model; patients with tumors bearing high methylation rates showed better survival within the first two to three years but poorer overall survival long term.

To assess the potential of DNA methylation as an independent prognostic marker, we further trained Cox regression models on all cancer types and included age, stage, and gender, when relevant. We found five cancers in which CIMP status provided an added value in improving accuracy of patient risk stratification: ACC (hazard ratio 4.4), HNSC (0.5), KIRP (6.8), LGG (0.3) and MESO (3.5) (Figure 4C and Supplemental Table 8). We interpret the results as CIMP positivity within ACC, KIRP and MESO tumors is associated with a worse prognosis, consistent with previous reports [25, 74, 82]. Meanwhile, the highly methylated tumors in HNSC and LGG are associated with a better prognosis, previously reported for LGG [28, 29] only. Finally, we reported for the first time that HNSC patients with highly methylated tumors were likely to have a good prognosis, independent of age, gender or stage (Figure 4A).

To illustrate the translation of our CIMP testing results to the clinic, we used a logistic regression (90%/10% training/test set split, balanced class weights, 5-fold cross validation) to identify a set of up to five probes that could differentiate CIMP and non-CIMP status with near perfect accuracy. We successfully classified patient samples with a 5-fold average adjusted-balanced accuracy (ABAC) of 0.989 [IQR (0.981–1.000)] (Supplemental Table 9). The performance on the held-out test set showed an average ABAC of 0.936 [IQR (0.900–1.000)]. We concluded that for most cancer types, we could test for the methylation status of up to five cancer probes in a cost-effective manner and thereby assess the CIMP status. Of note, *IDH1* mutation status was also a significant prognostic factor in LGG and GBM (although to a lesser extent than CIMP status) (Supplemental Figure 10) and could form the basis of a cost-effective clinical test.

### Pathway enrichment analysis identified nervous system development, pattern specification, cell signaling, differentiation and proliferation as potential downstream events of cancers with CIMP

We sought to identify downstream effects of hypermethylation by ascertaining which genes and pathways were ultimately affected. We defined potential downstream CIMP targets as genes that are both differentially expressed and associated with CGI hypermethylation in the high-methylation cluster.

**Figure 5:**
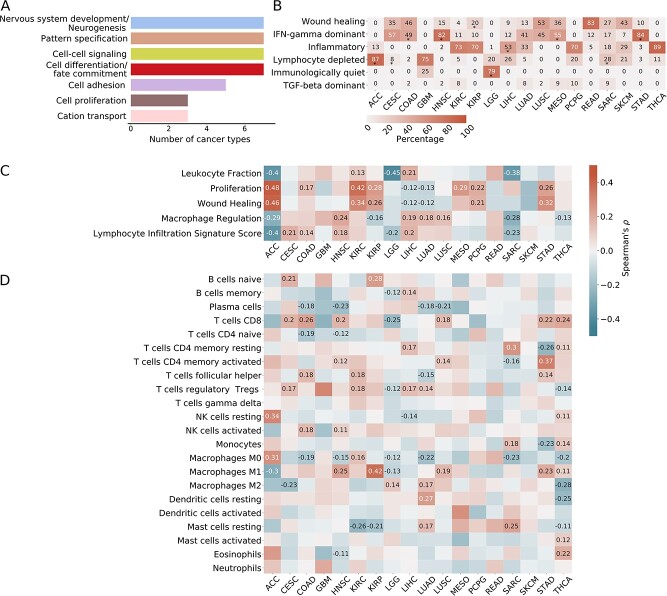
Downstream effects of CIMP and associations with immune cell composition. (**A**) Enriched pathways in the DAVID analysis. We selected genes that were both differentially expressed and presented hypermethylation in associated probes in the high-methylation group; we computed their enrichment in the DAVID [61] tool. The number of cancer types for which the pathways were enriched is indicated. (**B**) Enrichment in immune subtypes as described in Thorsson *et al*. [64]. Percentage of the immune subtypes in the high-methylation cluster is indicated. Subtypes significantly enriched in the high-methylation cluster as compared with the low-methylation cluster (Fisher’s *P* < 0.05) are indicated with a (^*^). (**C**) Spearman correlation coefficients between the CIMP score and the immune signatures and characteristics as described in Thorsson *et al*. [64]. (**D**) Spearman correlation coefficients between the CIMP score and the cell composition as precomputed using CIBERSORT [65]. Only significant associations (*P*-values adjusted with Benjamini–Hochberg correction <0.05) are annotated for (**C**) and (**D**).

The most enriched pathways for cancer-related events, reproducibly identified by the EASE score [61], were nervous system development/neurogenesis (7 of 19 cancers), pattern specification (7 of 19), cell–cell signaling (7 of 19) and cell differentiation/fate commitment (7 of 19), cell adhesion (5 of 19), cell proliferation (3 of 19) and cation transport (3 of 19). (Figure 5A, Supplemental Table 10). As nervous system development, developmental pathways, cell adhesion are all associated with cell migration and metastasis [87–89], DNA methylation could be a promising target for some cancer type-specific therapies.

### Tumors with CIMP show significant associations with specific immune subtypes

Turning to TME, we hypothesized that CIMP status might factor into a personalized medicine approach for some cancer types. Indeed, the immune activation of cancer types has been shown to correlate with classical therapies like cisplatin [90] or radiotherapy [91], as well as more recent therapies such as immune checkpoint inhibitors [92].

We computed the methylation enrichment of immune subtypes, as described by Thorsson *et al*. [64]. We also analyzed the correlation between CIMP score and TME characteristics [64], as precomputed by CIBERSORT [65] and xCell [66].

We observed several notable associations (Figure 5B): the high-methylation group was enriched in a specific immune subtype in 11 cancer types. Specifically, we observed associations between CIMP and the wound healing subtype in KIRP; the IFN-γ dominant subtype in COAD, stomach adenocarcinoma (STAD), HNSC and MESO; the inflammatory subtype in LIHC, the lymphocyte-depleted subtype in ACC and SARC and the immunologically quiet subtype in LGG.

Thorsson *et al*. [64] reported differences in prognosis linked to immune subtype. Due to its characteristic immunosuppressed microenvironment, the lymphocyte-depleted subtype conferred the worst prognosis. At the same time, the inflammatory subtype carried the best prognosis, consistent with the need for a dominant, type I immune response against cancer [93].

Our analysis confirmed the previously reported link between *IDH* mutations and lymphocyte depletion in LGG. The mechanism may be based on decreased leukocyte chemotaxis [94], as well as enrichment of the immunologically quiet subtype in highly methylated tumors of LGG [64].

We also found numerous significant associations between high-methylation and general immune signatures and characteristics (Figure 5C), namely association with significantly increased proliferation in seven cancer types and decreased proliferation in two. High methylation was also associated with increased macrophage regulation in four cancer types and decreased regulation in four, as well as a stronger lymphocyte infiltration in four and weaker in three.

Analysis of the immune composition deconvolved by CIBERSORT showed that some specific immune cell types were enriched in the high-methylation group (Figure 5D). Specifically, COAD, STAD and HNSC showed an increase in activated immune cells (dubbed, ‘immune hot’), whereas in LIHC, LUAD and SARC, enrichment was apparent in resting or regulatory immune cells (dubbed, ‘immune cold’). These observations were generally in agreement with the results of the immune composition analysis by xCell [66] (Supplemental Figure 11).

Overall, the high number of significant associations between the CIMP score and immune cell composition indicated the methylation status of cancerous cells may influence the TME, making CIMP a potential biomarker for immunotherapy in the clinics. However, more experimental work is needed to investigate the functional relationship between CIMP and immune cell composition in cancers.

## Discussion

Our goal was to define CIMP in human cancers and ascertain with available data whether the phenotype was present in all cancer types. Our primary aim was for the first time to create a definition agnostically (not based on a preexisting panel of genes or a priori knowledge of methylated positions, as had been done previously [11, 13, 17–19, 21, 22, 27, 30–32, 95]). The main advantage of our technique is its reliance on unbiased signals from as many informative probes as possible, while eliminating biases associated with gender, age at diagnosis, and tumor purity.

Based on CGI methylation patterns, we characterized 26 cancer types into two categories, CIMP-positive and negative and investigated the effect of dysregulated methylation on clinical outcome. We discovered CGI hypermethylation was significantly associated with survival in 8 of 19 CIMP-positive cancer types and had a prognostic value independent from age at diagnosis, stage, or gender in 5, including ACC, HNSC, KIRP, LGG and MESO.

We also have identified candidate driver events of CIMP in four broad categories: MSI, mutations in DNA demethylation genes, mutations in histone demethylation genes and mutations in upstream signaling pathways. We have investigated the potential downstream effects of CIMP and confirmed cellular functions known to be impacted by DNA methylation, such as cell–cell signaling, cell adhesion and neural system differentiation. We have also shed light on the link between CIMP and the TME, paving the way for potential further causal analysis.

Other studies have explored DNA methylation dysregulation in a pan-cancer manner [43–47]. However, our approach involved strict preprocessing of DNA methylation data, correcting for age, gender and tumor purity. In addition, we characterized CIMP status by scoring significant hypermethylation of CGIs in specific tumor subsets as compared with others within the same cancer type—as opposed to measuring generalized hypermethylation compared with normal tissue. For this reason, we did not screen for probes that were differentially expressed as compared with normal tissue; we only compared methylation events to normal tissue levels a posteriori (Figure 2E).

We acknowledge that our study does have limitations. For example, our definition of CIMP included not only CGIs but also shores and shelves, thereby excluding some cancer types, such as UCEC and esophageal carcinoma (ESCA), from the CIMP-positive category. Further, some cancer types are inherently age- or gender-related e.g. a better prognosis subgroup of younger patients has been documented for ESCA [96]. Although in our analysis we only discarded CpG probes as associated with age or gender when this correlation had been observed in at least two cancer types, our analysis in such cancers might still suffer from overfiltering. We estimate this effect being minor given similar proportions of filtered probes across all cancer types (Figure 2A). However, we cannot exclude that a less stringent correction for age- and gender-related effects might change the CIMP-negative status of some cancer types. Inversely, we did not correct for ethnicity, which might account for some portion of the variability in DNA methylation [97]. However, ethnicity did not correlate with the high-methylation group in any of the cancer types.

In addition, we compared the methylation patterns of cancerous samples to those of adjacent normal tissue, assuming (perhaps incorrectly) that the cancer originated from the same tissue of origin. We did not correct for genomic differences between individuals, even though methylation can be influenced by individual SNPs. Instead, we assumed that we circumvent the issue by using the Illumina 450 k array to obtain data from functional parts of the genome, which are less subject to variation [50]. For interpretability and comparability with other studies, we chose to use beta-values rather than M-values to characterize the level of DNA methylation, although M-values may have higher power for detecting differential methylation levels [98].

We also chose to use a hard assignment clustering algorithm instead of the soft assignment of points to clusters. Although the former technique has the advantage of eased analysis and interpretation, it introduces loss of information and potential inaccuracies in further analyses. We tried to mitigate this effect by introducing some measure of uncertainty and using only HC patients for subsequent analyses. This can, however, lead to overfiltering patients that could represent the complexity and diversity of the underlying biology of CIMP in cancer.

Finally, we simplified some analyses, which may have affected results. For example, we fixed the cutoff for CIMP presentation at an arbitrary value: 20% difference in average beta-values. To palliate the somewhat arbitrary nature of the cutoff to define a cancer type as CIMP-positive, we provide information on potential mutational drivers and the survival analysis for CIMP-negative cancers in Supplemental Materials (Supplemental Figures 12–14 and Supplemental Tables 11–12). In addition, we did not perform a grid search to optimize the hyperparameters of the Random Forest classifiers, which may have altered results. We focused on the discovery of impactful features rather than on the classification of tumors for further analysis. Finally, we did not verify the proportional hazards assumption in the Cox regression model, as it is often ‘untrue’ in medical settings [99]. Hence, we advise interpreting hazard ratios as a weighted average of the true hazard ratio [100].

The results of this study align with published research, validating well-documented genomic drivers of CIMP (i.e. mutations in *IDH1/2* and *SETD2*). Noteworthily, we find that *IDH1* and *SETD2* mutations are potential shared drivers across nine CIMP-positive cancer types, albeit in sometimes rare subpopulations (such as in LUAD).

Similarly, our survival analysis confirmed for many cancer types previous reports of significant differences in survival linked to CIMP status [23, 25, 26, 28, 29, 74, 82–84]. We found that several of the genes discovered as mutated in the high-methylation group were known tumor suppressor genes (e.g. *TP53, ATRX, NF1*) or oncogenes (e.g. *KRAS, BRAF, EGFR*). Although the relationship between some of these mutations and CIMP has been investigated (e.g. for BRAFV600 [101] or PIK3CA [102]), studies on the causality between DNA hypermethylation and activation or inactivation of these genes are still lacking. Understanding the link between DNA hypermethylation and genomic variants in these oncogenes and tumor suppressors would potentially enable better targeted therapy in the affected cancer types.

We note that we could not find candidate driver events for PC and thyroid carcinoma (THCA), suggesting either lack of statistical power or heterogeneous mechanisms. In addition, we could not reproduce results that linked the *SDHx* gene family mutations to CIMP in PCPG [103].

In terms of survival (correcting for age at diagnosis, clinical stage and gender), only GBM and LGG [29] were previously analyzed using a similar multivariate analysis. In contrast, we report CIMP-linked survival differences for HNSC, both univariate and multivariate analyses, and SKCM, previously reported as mixed [83–86].

In terms of clinical relevance, we showed the ability to cost-effectively predict with near perfect accuracy the CIMP status of almost all CIMP-positive cancer types using up to five probes. This predictive factor can be useful to stratify patients, for instance, using CIMP status as a more accurate survival prognosticator than *IDH1* status for patients with LGG and GBM.

There were some cancer types with reported CIMP that we did not identify as CIMP-positive (bladder, breast, esophageal and UCEC). In addition, we could not reproduce previous associations of CIMP with clinical outcomes for KIRC [17], LUAD [19], STAD [13], LUSC [19] or SARC [104]. We note that most of these reports used gene panels to define CIMP, and the entire basis of this study was to provide an independent agnostic means to define the phenotype.

Also noteworthy, we were unable to reproduce previous results demonstrating the prognostic impact of CIMP on survival for COAD [105, 106]. We argue that this is not surprising, given that such reports showed mixed results [106] i.e. discussing the necessity of both *MSS* and *KRAS/BRAF* mutations to link CIMP status to survival [107].

Finally, we found numerous associations between CIMP status and the TME. Understanding how the methylation state of cancerous cells influences the tumor immunogenicity and microenvironment or vice versa warrants further investigation, as it might enable better prediction of the response to classical and immunotherapies of patients with different methylation states.

In conclusion, we have thus investigated and characterized the presence of CIMP in 26 cancer types using the TCGA database, highlighting that although CGI dysregulation is present in all studied cancer types, its level varies greatly cancer by cancer. We have shown substantial differences between CIMP and non-CIMP groups, mainly involving mutations and the altered expression of genes involved in DNA or histone methylation and demethylation. Finally, we have evidenced the biological and clinical importance of CIMP in predicting survival, finding significant differences in survival between the low- and high-methylation groups in eight cancer types overall and five specifically, after correction by age, stage and gender.

We have further exemplified the translational capability of methylation testing in the clinic with the use of a small panel that accurately predicts CIMP status. We have also investigated the potential immunomodulatory role of CIMP through immune subtype classification and immune cell correlation. Provided that several drugs targeting DNA methylation have been already approved for clinical use [108], we argue that elucidating the etiology of DNA methylation dysregulation in cancer, as well as understanding its impact on patient survival, would enable significant inroads in cancer treatment.

## Data Availability

The code used to perform the analysis and supplemental information on patient and cancer levels are available at https://github.com/BoevaLab/CIMP_etiology_oncogenic_transformation.

The TCGA datasets were derived from sources in the public domain at UCSC Xena browser: http://xena.ucsc.edu/.

The normal data for ACC and LAML are available in the Gene Expression Omnibus (GEO) dataset at https://www.ncbi.nlm.nih.gov/gds, and can be accessed with unique identifiers GSE77871 and GSE32149.

## Acronym section

ABAC: adjusted balanced accuracy; ACC: adrenocortical carcinoma; BLCA: Bladder Urothelial Carcinoma; BRCA: breast invasive carcinoma; CESC: cervical squamous cell carcinoma and endocervical adenocarcinoma; CI: confidence interval; CGI: CpG Island; CIMP: CpG island methylator phenotype; COAD: colon adenocarcinoma; DAVID: database for annotation, visualization and integrated discovery; ESCA: esophageal carcinoma; GBM: glioblastoma multiforme; HC patient: high confidence patient; HNSC: head and neck squamous cell carcinoma; HR: hazard ratio; KIRC: kidney renal clear cell carcinoma; KIRP: kidney renal papillary cell carcinoma; LGG: brain lower grade glioma; LAML: acute myeloid leukemia; LIHC: liver hepatocellular carcinoma; LR: logistic regression; LUAD: lung adenocarcinoma; LUSC: lung squamous cell carcinoma; MESO: mesothelioma; mRNA: messenger RNA; MSI: microsatellite instability; MSS: microsatellite stability; NS: non-significant; PAAD: pancreatic adenocarcinoma; PCPG: pheochromocytoma and paraganglioma; PRAD: prostate adenocarcinoma; READ: rectum adenocarcinoma; SARC: sarcoma; SD: standard deviation; SKCM: skin cutaneous melanoma; SSC: sample silhouette coefficient; STAD: stomach adenocarcinoma, TCGA: The Cancer Genome Atlas; THCA: Thyroid carcinoma; THYM: thymoma; TSS: transcription start site; UCEC: uterine corpus endometrial carcinoma.

**Key Points**

- To define cancer types characterized by CIMP, we analyzed CGI methylation, eliminating biases linked to age at diagnosis, gender, and tumor purity.
- Although consistent methylation dysregulation exists in all cancers, CIMP does not seem to be present in all cancer types studied.
- Mechanisms causing CIMP are heterogeneous, including mutations in *IDH1/2* and *SETD2* that were previously reported in specific cancer types, as well as reported for the first time here in new cancer types; the novel overexpression of *BORIS/CTCFL* spanned several cancer types.
- CIMP is often a prognostic factor: it influences survival in eight cancer types and is a prognostic marker independent of age at diagnosis, stage and gender for five cancers. This relationship was reported for HNSC for the first time in this study.
- CIMP appears to be linked to a specific TME in many cancer types, affecting immune cell composition and signatures.

## Supplementary Material

Supplemental_Materials_revised_final_bbab610

## Notes

**Josephine Yates** is a PhD candidate at ETH Zurich, Computer Science Department, Institute for Machine Learning, Zurich, Switzerland. Her interests are the epigenetic and transcriptional heterogeneity of cancer cells and its relationship to the efficiency of treatments.

**Valentina Boeva** is a professor at ETH Zurich, Computer Science Department, Institute for Machine Learning, Zurich, Switzerland. Her research focuses on cancer epigenetics, computational biology and machine learning.
