# Supplementary material for: Deciphering the etiology and role in oncogenic transformation of the CpG island methylator phenotype: a pan-cancer analysis
Source: Brief Bioinform. 2022 Feb 2;23(2):bbab610. doi: 10.1093/bib/bbab610 (PMC8921629; doi:10.1093/bib/bbab610)
Supplement: Supplemental_Materials_revised_final_bbab610 [file supplemental_materials_revised_final_bbab610.zip › Supplemental_Materials_revised_final_bbab610.docx]

## Supplemental Figures


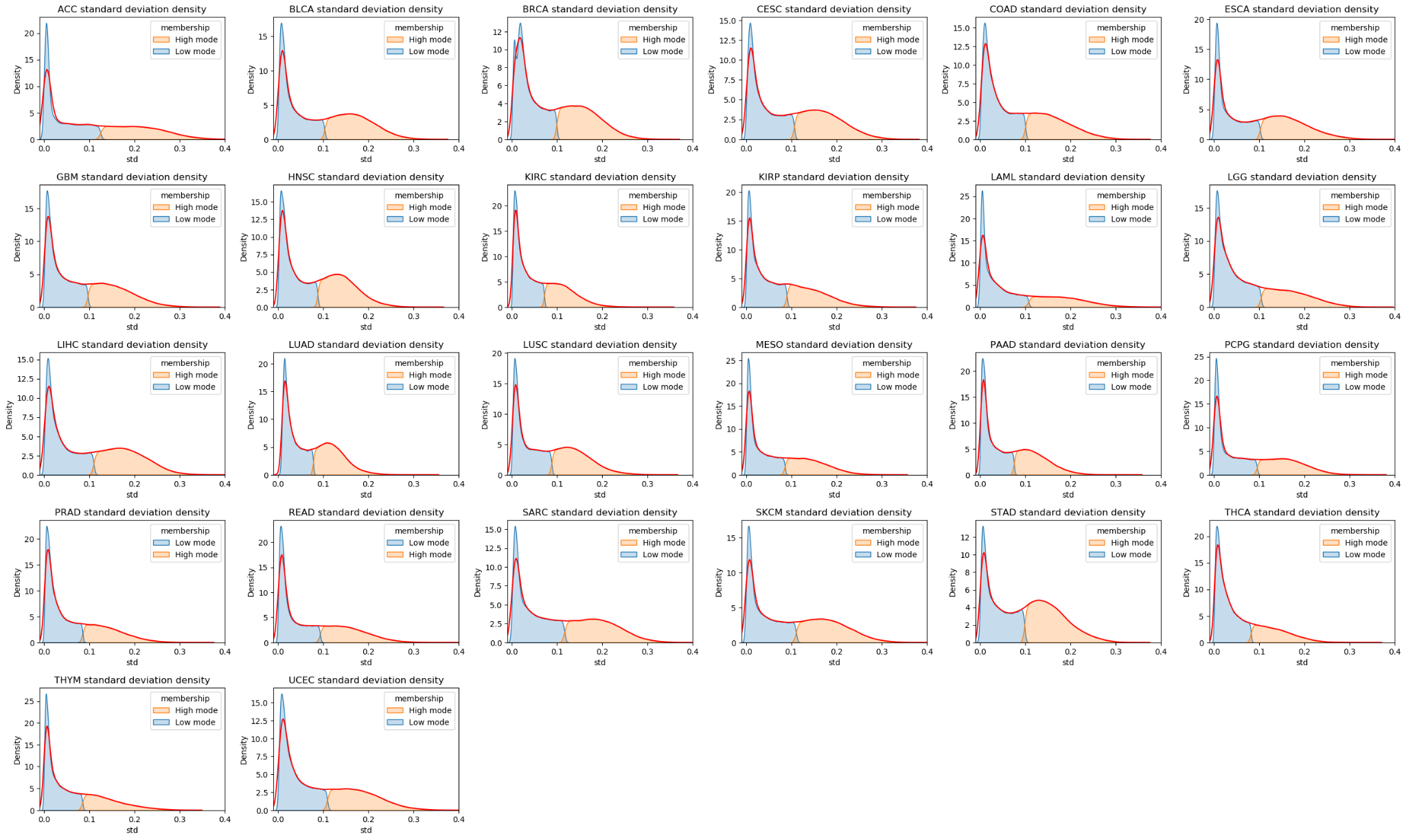


**Supplemental Figure 1: Selection of highly variable probes in 26 cancer types (TCGA) based on *k-*means clustering of standard deviation (SD) of probe beta-values.** The SD values were computed for each probe and the associated densities are represented as a red envelope. *K*-means clustering (*k* = 2) was performed on SD values and the groups were assigned high mode or low mode depending on their average SD. The low mode probes were removed from further analysis.


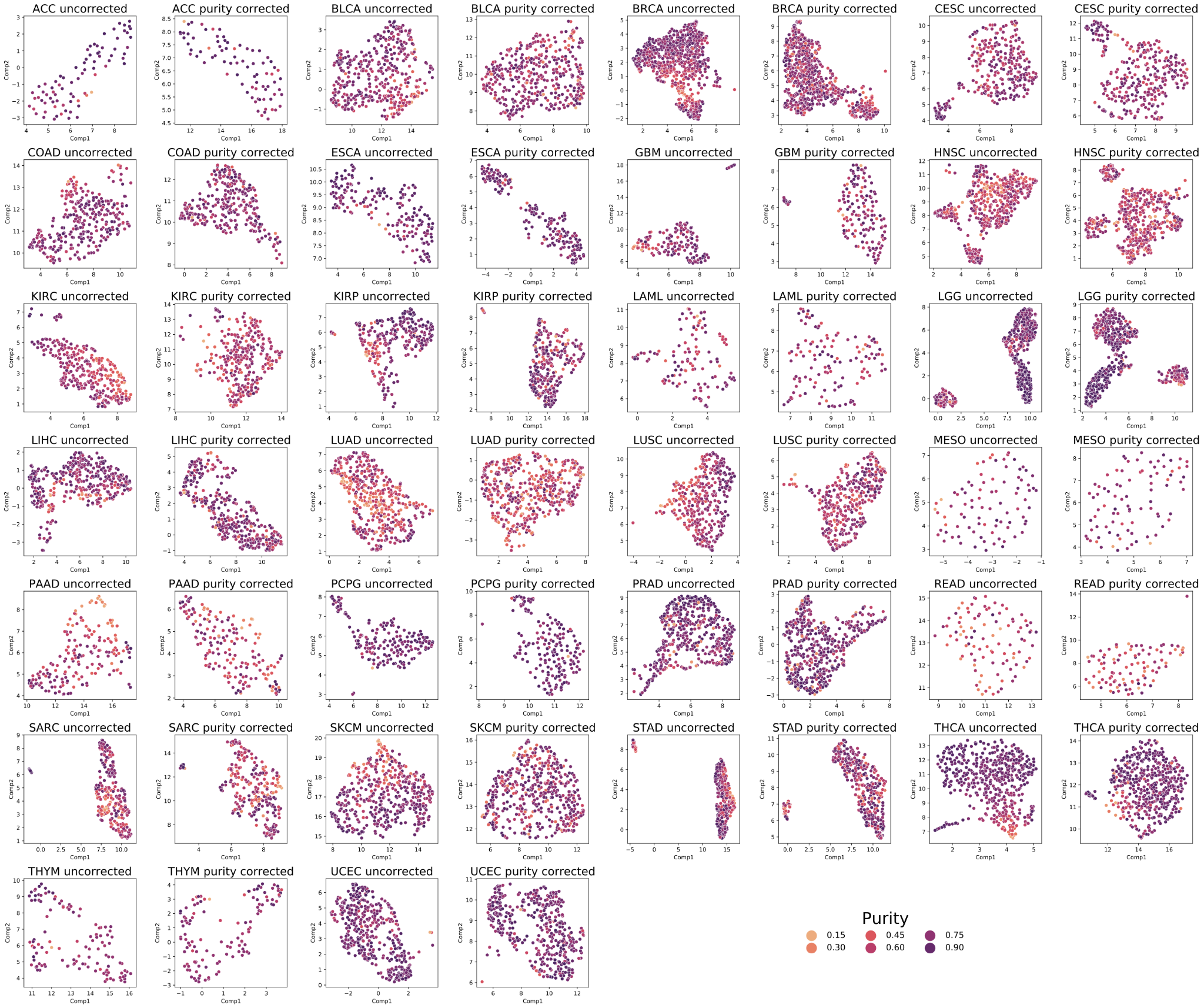


**Supplemental Figure 2: Correction for tumor purity prevents artificial clustering of samples with similar purity values.** The UMAP embeddings of samples before and after purity correction for 26 cancer types (Methods).


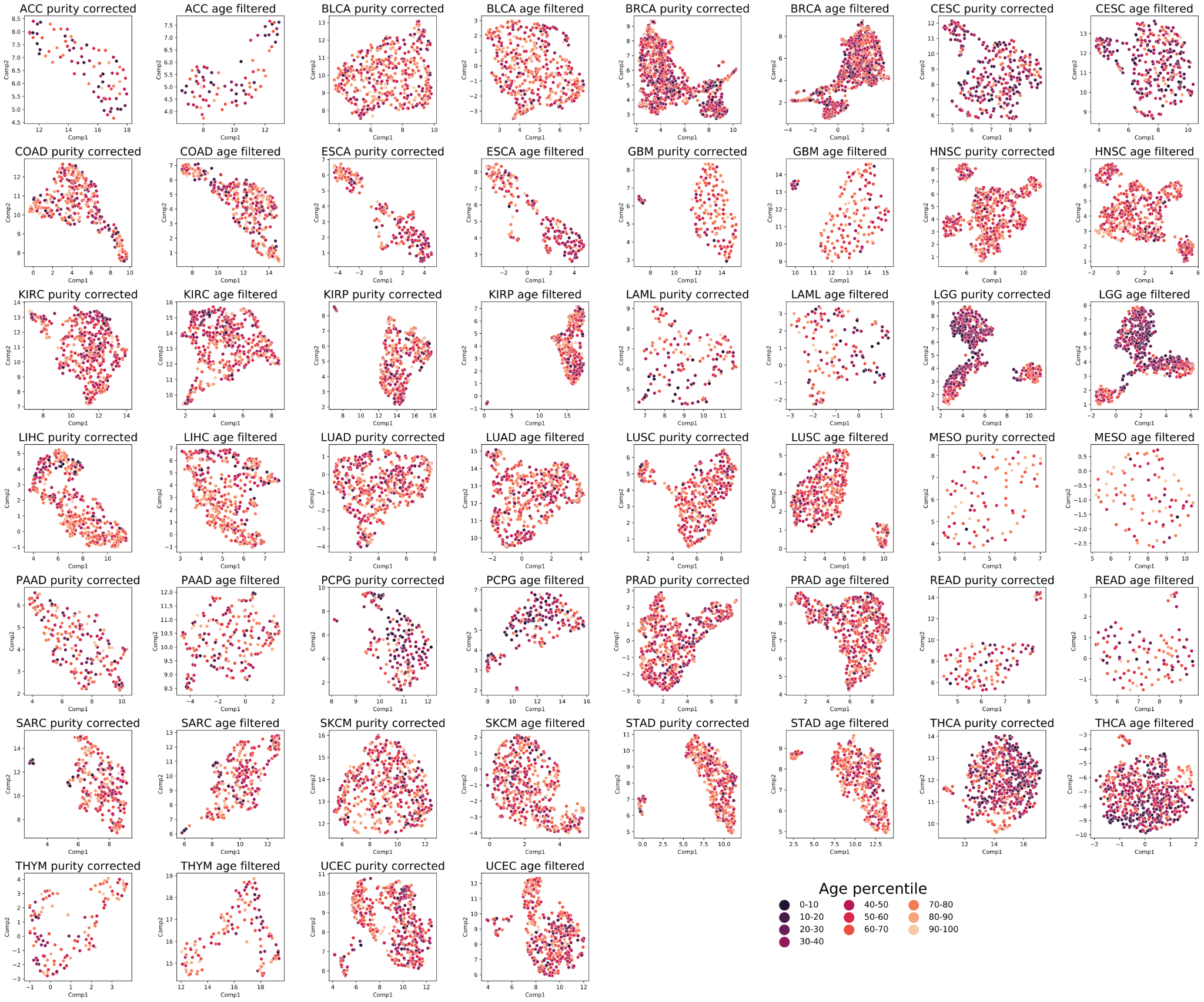


**Supplemental Figure 3: UMAP representation of samples before and after age-related probe filtering for 26 cancer types.** The UMAP embeddings were computed after purity correction but before filtering of age-related probes (left) and after purity correction and filtering of age-related probes (right).


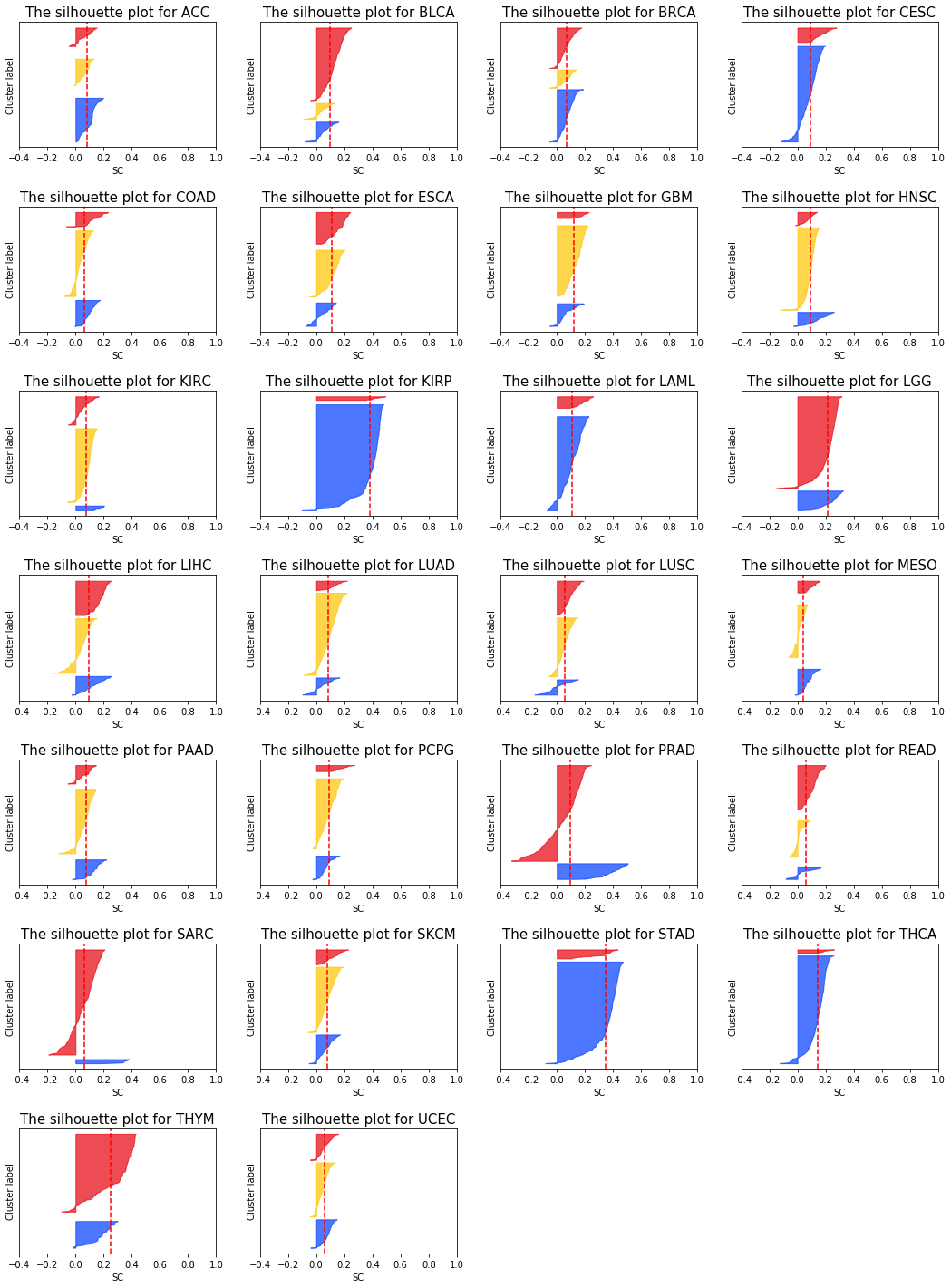


**Supplemental Figure 4: Silhouette coefficients (Euclidean distance) for each patient for 26 cancer types representing the quality of clustering.** Bars representing tumor samples are colored according to their cluster membership (blue: low-methylation group, yellow: intermediate-methylation group, red: high-methylation group). The average silhouette coefficient is indicated as a red dotted line.

**
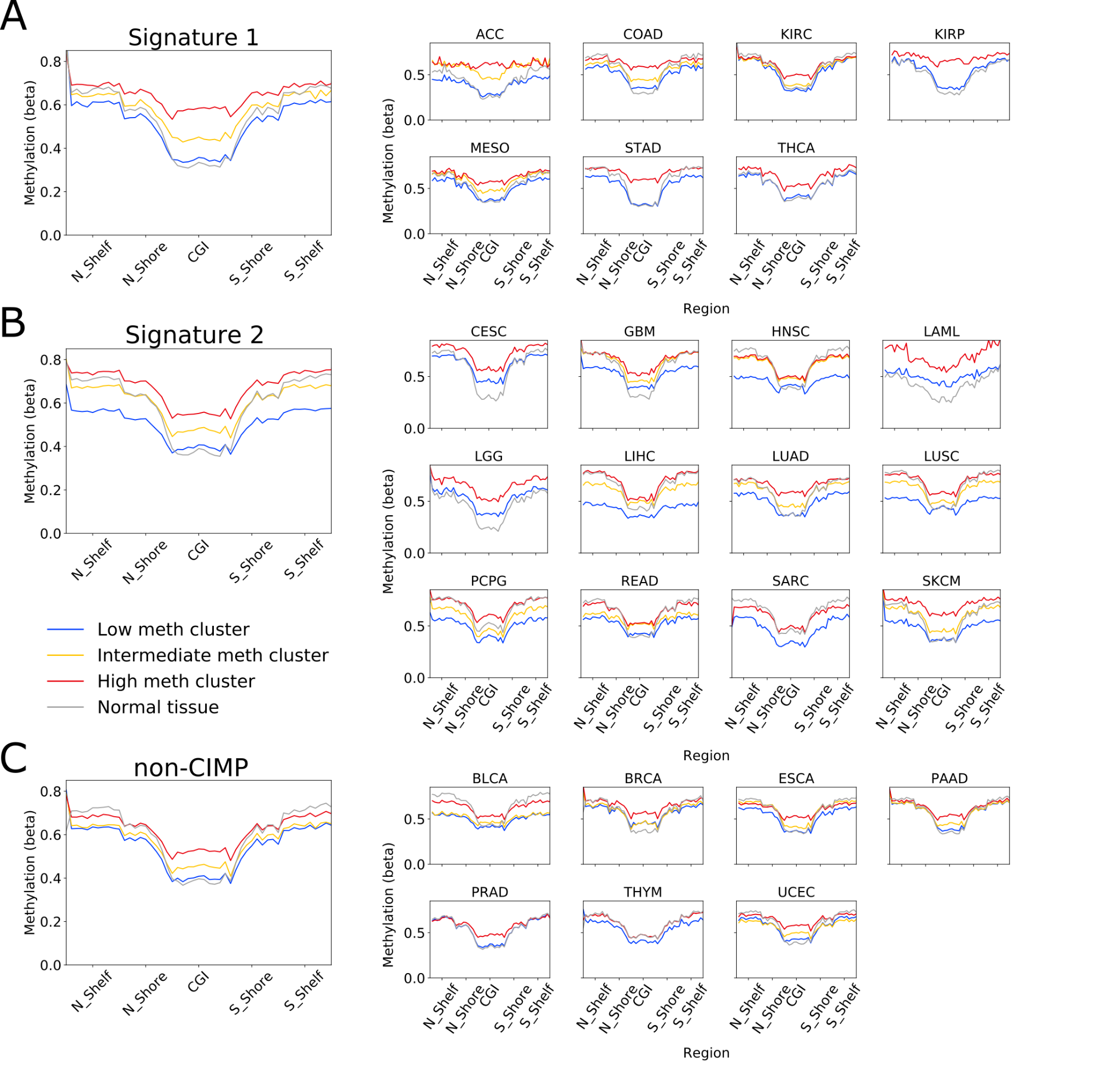
Supplemental Figure 5: Average methylation profiles over CpG Islands, shores and shelves for 26 cancer types for CIMP signature 1 (A), CIMP signature 2 (B) and non-CIMP cancers (C).** The north shelves (N_shelf), north shores (N_shore), CpG Islands (CGI), south shore (S_shore) and S_shelves (S_shelf) are broken into 10 bins each. (**A**) Signature 1 consists of no or small hypermethylation of shores and shelves with relatively higher hypermethylation of CGI; (**B**) Signature 2 represents global hypermethylation with equal or relatively higher increase of DNA methylation in shores and shelves; (**C**) Non-CIMP cancer types show little gain in DNA methylation over CGIs, shelves and shores methylation.


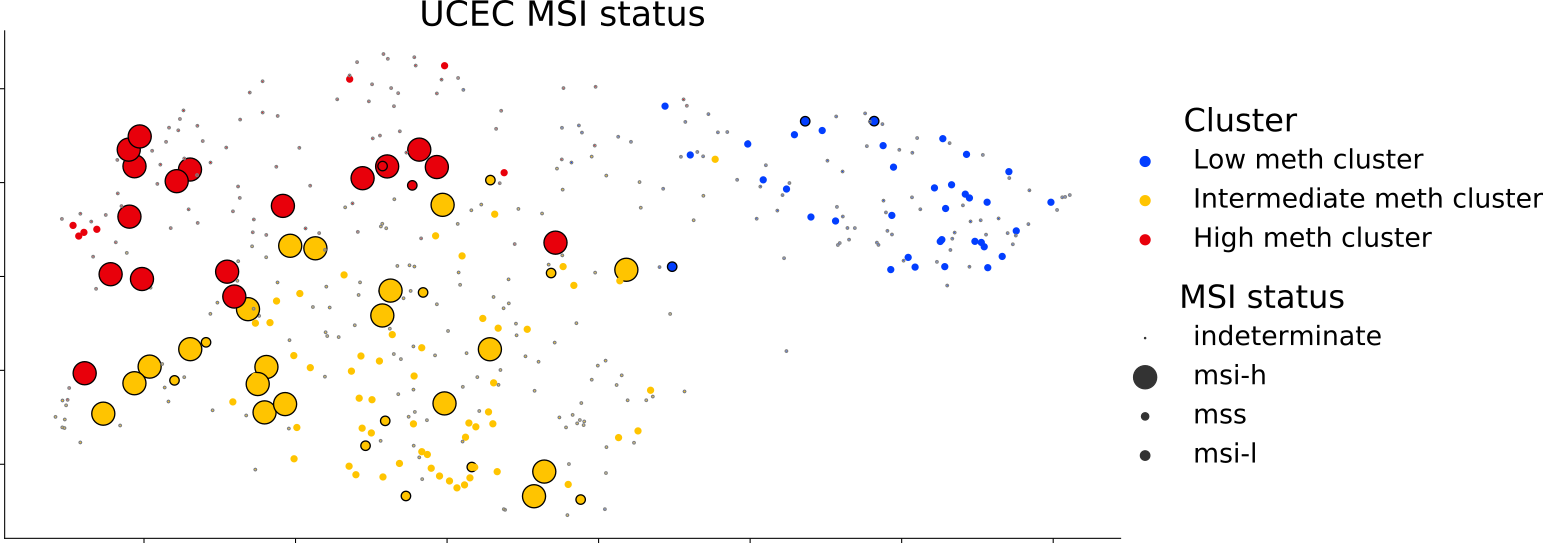


**Supplemental Figure 6: UMAP representation of the MSI status in UCEC.** Patients are represented with a two-dimensional UMAP embedding and are colored according to their cluster membership. Patients are sized according to their microsatellite instability status (msi-h: MSI high, mss: microsatellite stable, msi-l: MSI low).


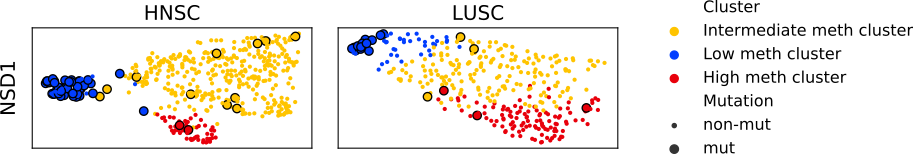


**Supplemental Figure 7: UMAP representation of the *NSD1* mutation status in COAD, HNSC and LUSC.** Patients are represented with a two-dimensional UMAP embedding and are colored according to their cluster membership. Patients presenting a mutation in the *NSD1* gene are indicated by large circles.


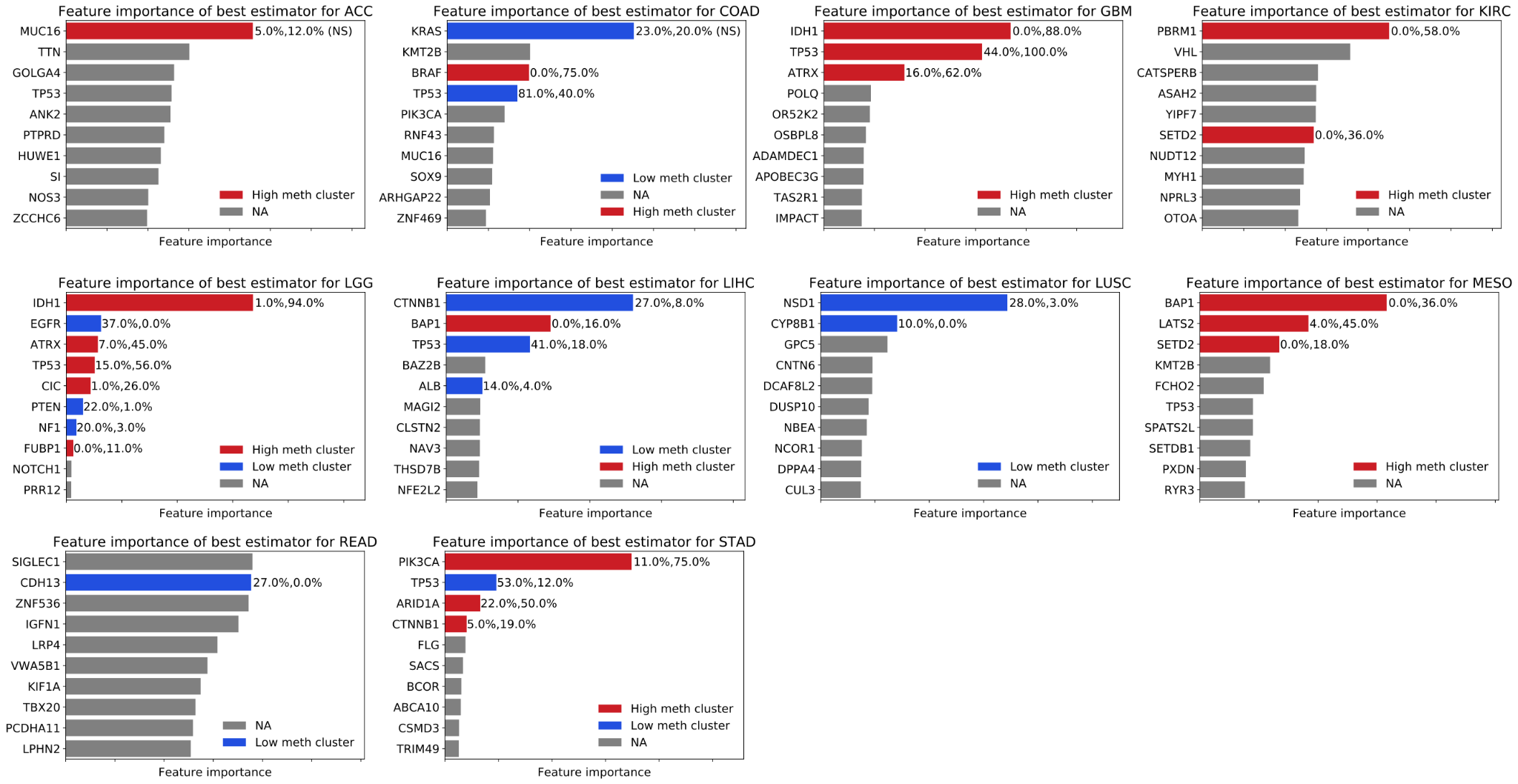


**Supplemental Figure 8: Feature usage for the Random Forest classifiers identifies potential genomic drivers of CIMP in 10 cancer types (ranking shown for 10 cancer types in which Random Forest outperformed random classifiers).** A Random Forest model was trained on all non-silent mutations for each cancer type. Cancer types with an adjusted balanced accuracy (ABAC) above zero and persistence of selected features in the top 10 most useful features over 10 fold cross validation were selected. Features are ranked by importance, bars are colored according to what methylation group was enriched in the given mutation (high or low methylation group; NA is used when the feature was not selected among the top 10 features across all 10 folds). Percentage of patients presenting the mutation in a given group (low-methylation group %, high-methylation group %) are given next to the bars. Mutations that do not pass the 0.1 threshold for the Benjamini Hochberg corrected Fisher exact test *p*-value are indicated by non-significant (NS).


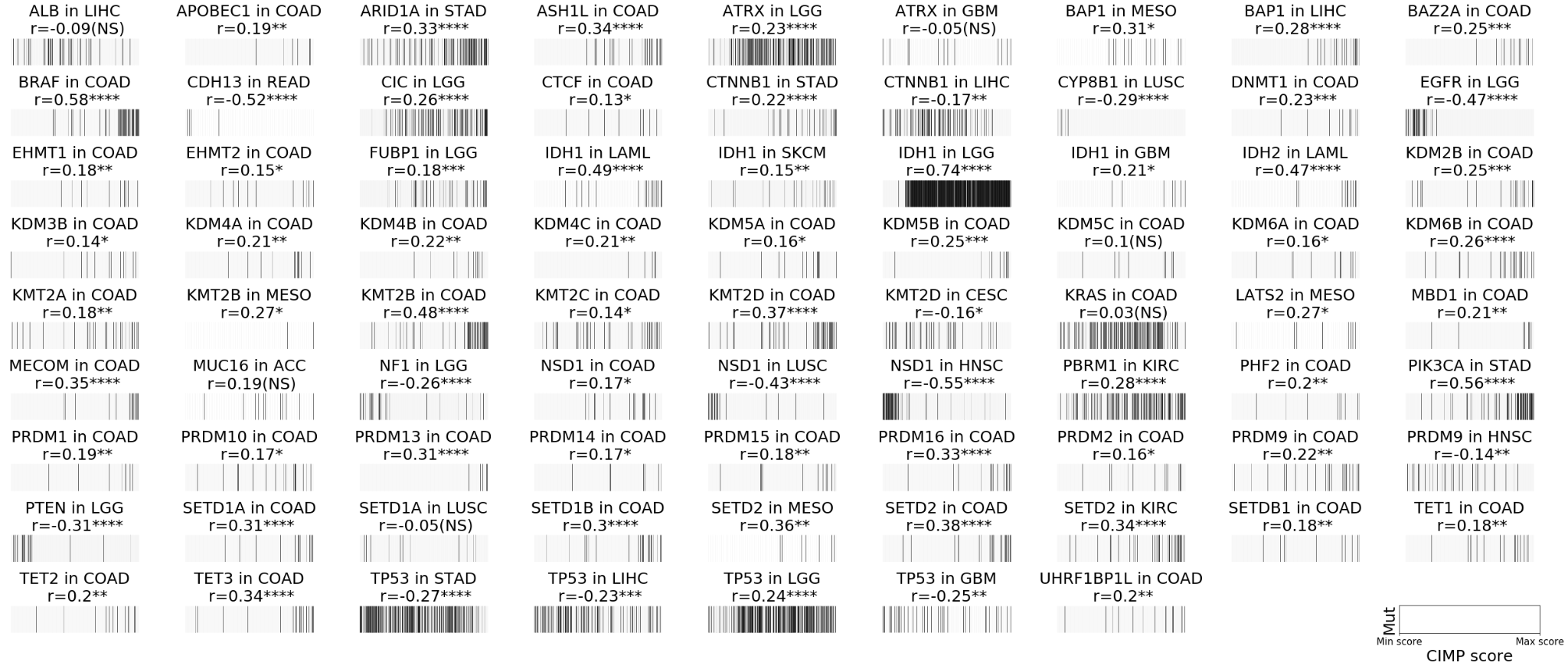
**Supplemental Figure 9: Significantly correlated mutations versus CIMP score.** Patients are ranked for each cancer type according to their CIMP score. Patients presenting a mutation in the gene of interest are indicated by a black bar. Only significant mutations (Fisher exact test *p*<0.05) were reported. Significance is reported for FDR Benjamini-Hochberg corrected *q*. NS: *q*>0.1, *: 0.01≤*q*<0.1, **: 0.001≤*q*<0.01, ***: 0.0001≤*q*<0.001, ****: *q*<0.0001.


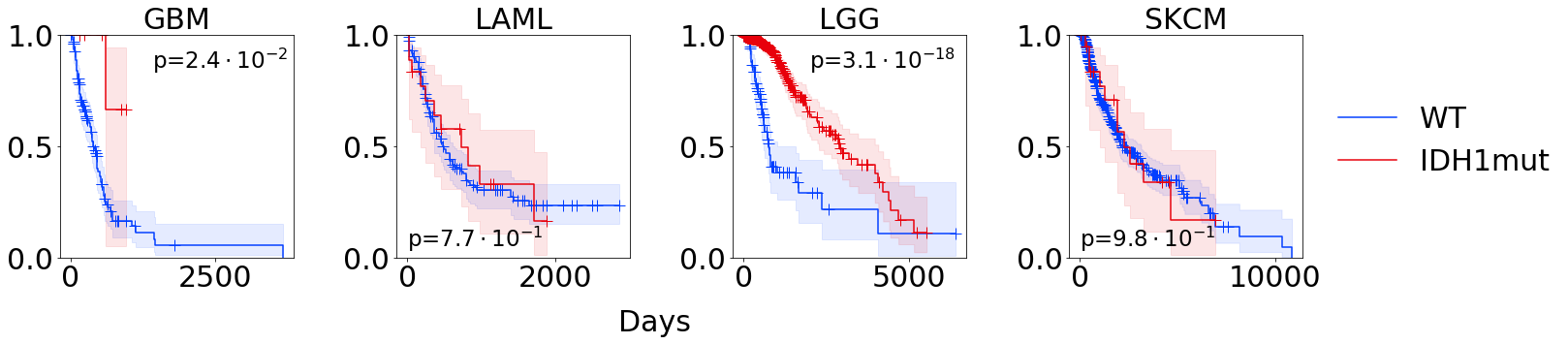


**Supplemental Figure 10: Kaplan-Meier representation of survival between groups presenting IDH1 mutations and wild type (WT) for 4 cancer types.** In the 4 cancer types presenting a significant enrichment in IDH1 mutations in the high-methylation group, we stratified patients according to their *IDH1* mutation status. We then computed the log-rank test associated with the difference in survival between the two groups.


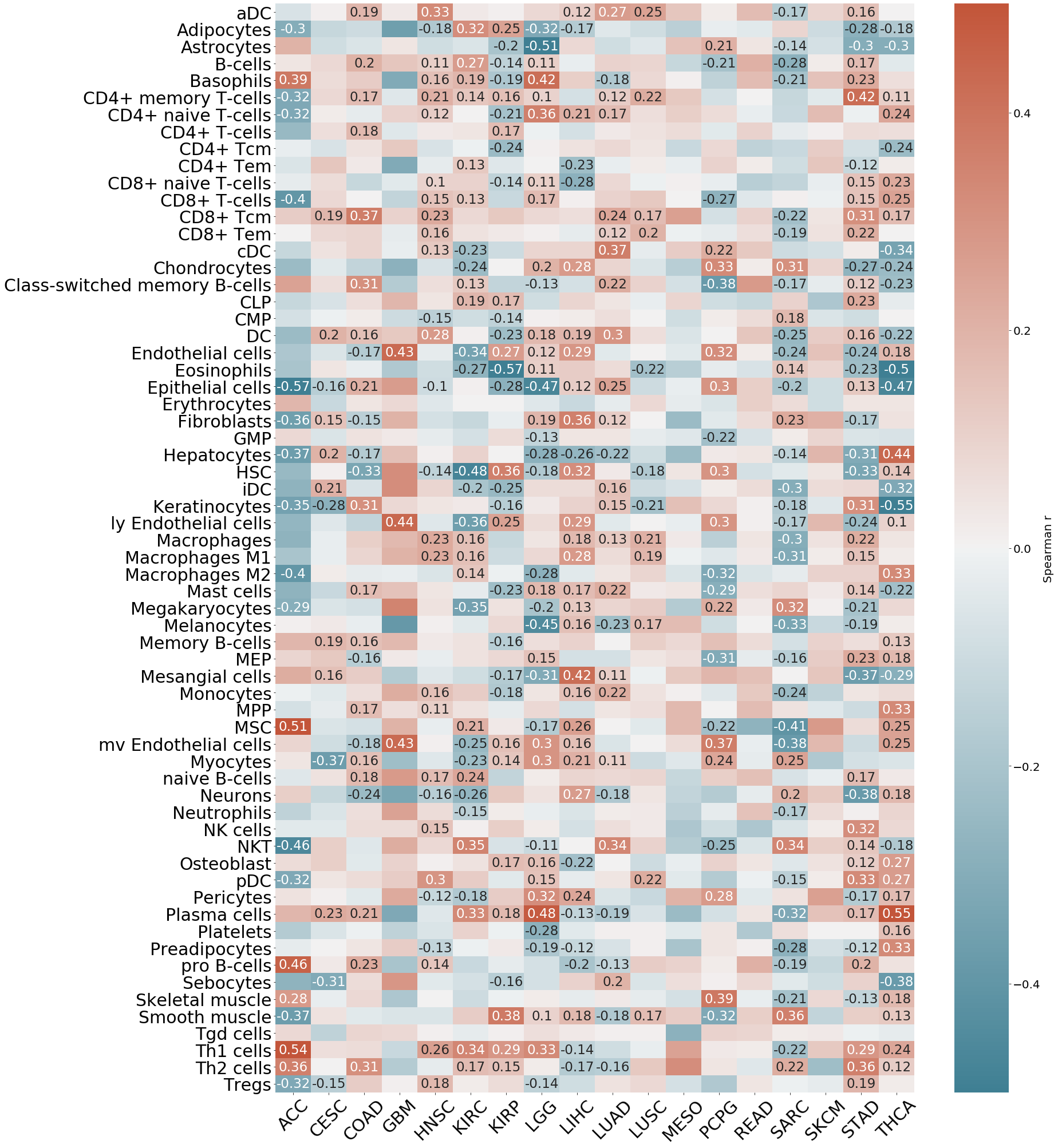


**Supplemental Figure 11: Spearman correlation coefficients between the CIMP score and the cell composition as pre-computed using xCell** [[66]](https://paperpile.com/c/NzlxcQ/dnQEq). Only significant associations (*p*-values adjusted with Benjamini-Hochberg correction < 0.05) are annotated.


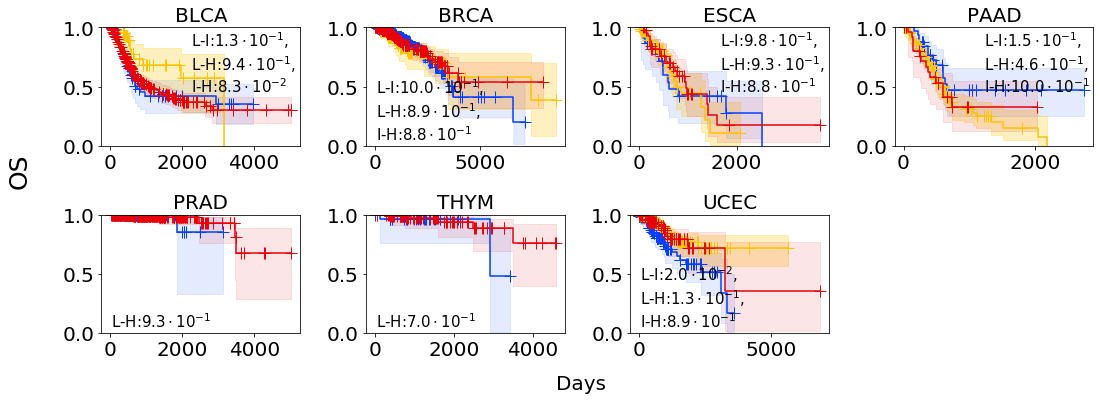


Supplemental Figure 12: Kaplan-Meier representation of univariate overall survival (OS) analysis for seven CIMP-negative cancer types. 95% confidence interval (CI) is represented by a colored area around the Kaplan-Meier curve. The associated log-rank test *p*-value is indicated as low- versus high-methylation group (L-H), and when relevant, low versus intermediate (L-I) and intermediate versus high-methylation groups (I-H).


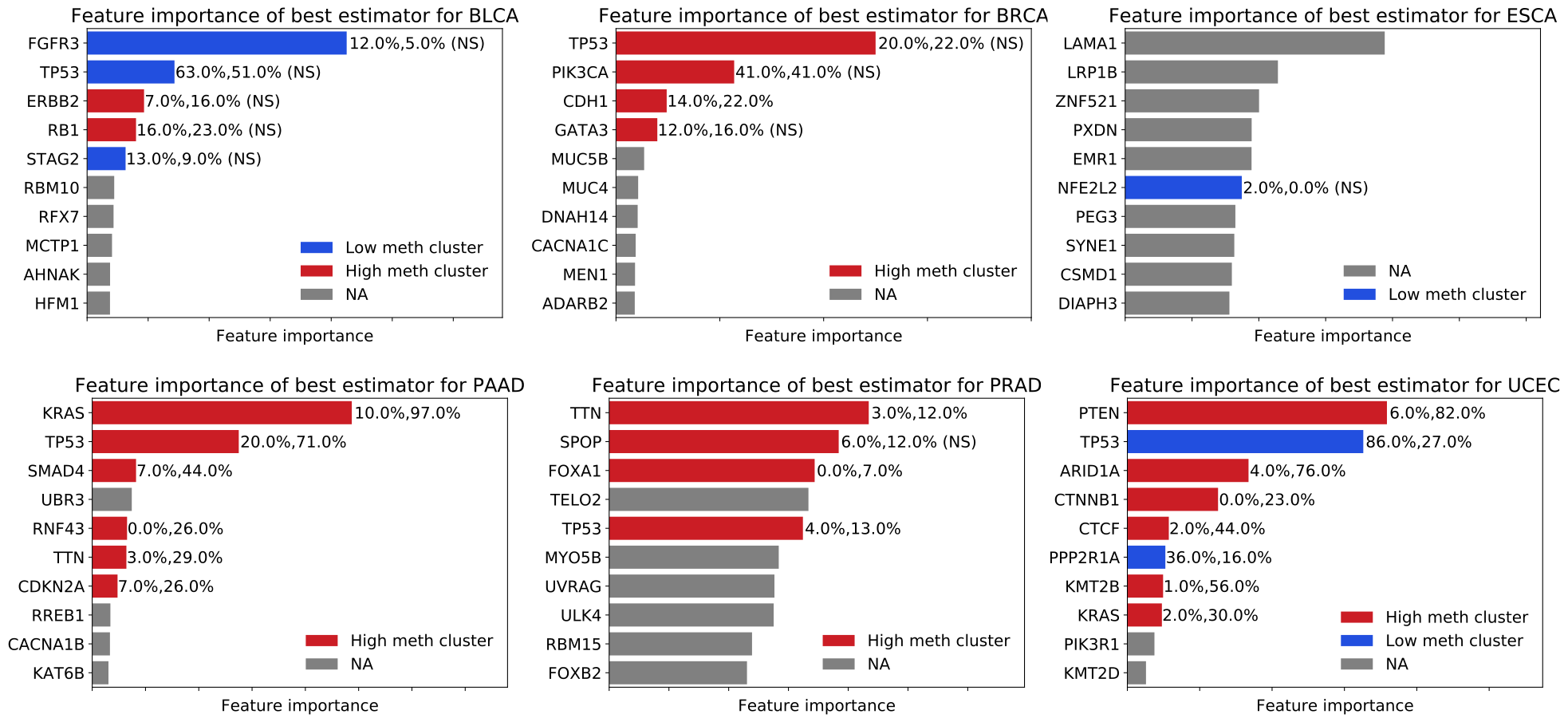


Supplemental Figure 13: Feature usage for the Random Forest classifiers identifies potential genomic drivers of the high methylation group in 6 CIMP-negative cancer types (ranking shown for 6 cancer types in which Random Forest outperformed random classifiers). A Random Forest model was trained on all non-silent mutations for each cancer type. Cancer types with an adjusted balanced accuracy (ABAC) above zero and persistence of selected features in the top 10 most useful features over 10 fold cross validation were selected. Features are ranked by importance, bars are colored according to what methylation group was enriched in the given mutation (high or low methylation group; NA is used when the feature was not selected among the top 10 features across all 10 folds). Percentage of patients presenting the mutation in a given group (low-methylation group %, high-methylation group %) are given next to the bars. Mutations that do not pass the 0.1 threshold for the Benjamini Hochberg corrected Fisher exact test *p*-value are indicated by non-significant (NS).


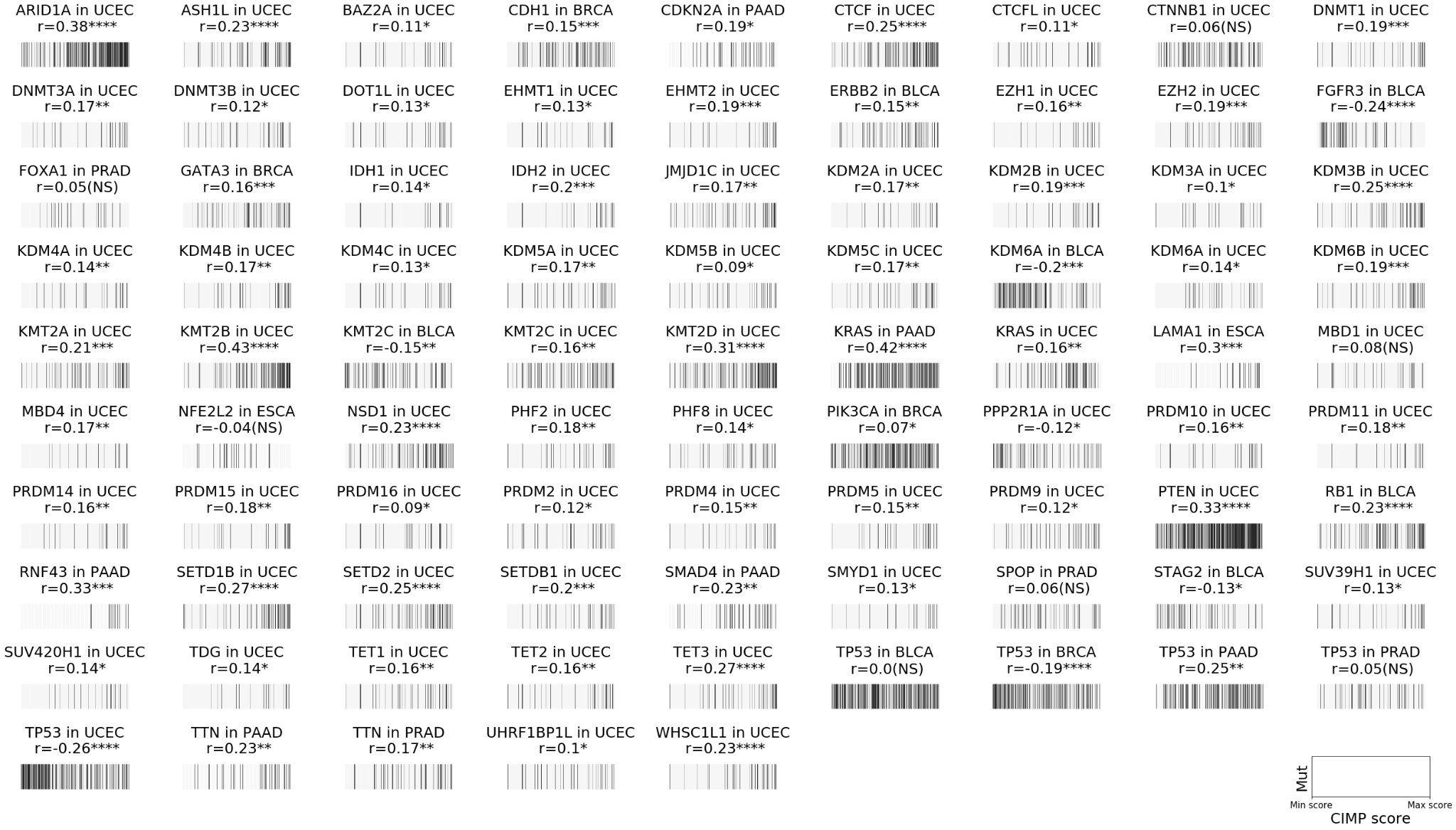


Supplemental Figure 14: Significantly correlated mutations versus CIMP score for CIMP-negative cancer types. Patients are ranked for each cancer type according to their CIMP score. Patients presenting a mutation in the gene of interest are indicated by a black bar. Only significant mutations (Fisher exact test *p*<0.05) were reported. Significance is reported for FDR Benjamini-Hochberg corrected *q*. NS: *q*>0.1, *: 0.01≤*q*<0.1, **: 0.001≤*q*<0.01, ***: 0.0001≤*q*<0.001, ****: *q*<0.0001.

Supplemental Tables

Supplemental Table 1: Comparison of average silhouette score for 26 studied cancer types in TCGA for 5 clustering methods. The number of clusters corresponds to that used in the main paper (provided in Supplemental Table 2). Agglomerative clustering is performed with Euclidean distance and Ward linkage. *K*-means is performed with *K*-means++ initialization. Gaussian Mixture Models are computed with diagonal covariance matrices on the UMAP reduced space of 5 dimensions. Louvain is computed on the Manhattan distance affinity matrix with resolution tuned to obtain the optimal number of clusters. The silhouette coefficient is computed in the full dimensional space using Euclidean distance.

| **Cancer type** | **Spectral** | **Agglomerative** | ***K*-means** | **Gaussian Mixture Model** | **Louvain** |
| --- | --- | --- | --- | --- | --- |
| **ACC** | 0.081 | 0.066 | 0.071 | 0.038 | 0.061 |
| **BLCA** | 0.099 | 0.025 | 0.054 | 0.059 | 0.103 |
| **BRCA** | 0.070 | 0.066 | 0.072 | 0.068 | 0.062 |
| **CESC** | 0.091 | 0.071 | 0.081 | 0.078 | 0.039 |
| **COAD** | 0.062 | 0.054 | 0.064 | 0.045 | 0.031 |
| **ESCA** | 0.111 | 0.091 | 0.127 | 0.099 | 0.123 |
| **GBM** | 0.122 | 0.063 | 0.107 | 0.022 | 0.083 |
| **HNSC** | 0.094 | 0.062 | 0.073 | 0.053 | 0.002 |
| **KIRC** | 0.079 | 0.085 | 0.085 | 0.049 | 0.066 |
| **KIRP** | 0.382 | 0.258 | 0.265 | 0.381 | 0.259 |
| **LAML** | 0.111 | 0.141 | 0.125 | 0.070 | 0.087 |
| **LGG** | 0.220 | 0.216 | 0.200 | 0.215 | 0.187 |
| **LIHC** | 0.095 | 0.118 | 0.107 | 0.078 | 0.072 |
| **LUAD** | 0.087 | 0.068 | 0.061 | 0.034 | 0.015 |
| **LUSC** | 0.056 | 0.047 | 0.055 | 0.033 | 0.004 |
| **MESO** | 0.041 | 0.054 | 0.074 | 0.030 | 0.076 |
| **PAAD** | 0.073 | 0.082 | 0.072 | 0.061 | 0.105 |
| **PCPG** | 0.089 | 0.061 | 0.047 | 0.049 | 0.047 |
| **PRAD** | 0.093 | 0.085 | 0.115 | 0.085 | 0.100 |
| **READ** | 0.058 | 0.027 | 0.058 | 0.030 | 0.031 |
| **SARC** | 0.066 | 0.111 | 0.109 | 0.042 | 0.104 |
| **SKCM** | 0.082 | 0.079 | 0.074 | 0.060 | 0.024 |
| **STAD** | 0.345 | 0.345 | 0.271 | 0.135 | 0.124 |
| **THCA** | 0.142 | 0.034 | 0.112 | 0.125 | 0.039 |
| **THYM** | 0.249 | 0.277 | 0.302 | 0.241 | 0.014 |
| **UCEC** | 0.057 | 0.053 | 0.064 | 0.053 | 0.037 |
| **Avg (IQR)** | **0.117 [0.071-0.111]** | **0.101 [0.056-0.106]** | **0.109 [0.066-0.114]** | **0.086 [0.043-0.084]** | **0.073 [0.033-0.102]** |

Supplemental Table 2: Statistics on analyzed cancer types. We report the number of samples analyzed, number of CpG ids after filtering, number of optimal clusters, number of significantly differentially expressed CpG ids between clusters, Kruskal-Wallis (Bonferroni corrected) *p*-value of significance of the distribution of average beta-values between clusters (L-H: low vs high, L-I: low vs intermediate, I-H: intermediate vs high) and percentage of patients with a positive silhouette coefficient (High Confidence (HC) patients).

| **Cancer type** | **Sample #** | **CpG ids #** | **Cluster #** | **Significant CpG ids #** | **KW p** | **HC patients (%)** |
| --- | --- | --- | --- | --- | --- | --- |
| **ACC** | 80 | 31,099 | 3 | 3,200 | L-H: <10^-10,^ L-I: <10^-10^ , I-H: <10^-10^ | 95 |
| **BLCA** | 413 | 32,717 | 3 | 22,024 | L-H: <10^-10,^ L-I: <10^-10^ , I-H: <10^-10^ | 93 |
| **BRCA** | 787 | 23,506 | 3 | 19,318 | L-H: <10^-10,^ L-I: <10^-10^ , I-H: <10^-10^ | 94 |
| **CESC** | 309 | 30,370 | 2 | 7,188 | L-H: <10^-10,^ L-I: 0.67 , I-H: <10^-10^ | 89 |
| **COAD** | 298 | 25,213 | 3 | 11,663 | L-H: <10^-10,^ L-I: <10^-10^ , I-H: <10^-10^ | 85 |
| **ESCA** | 186 | 32,421 | 3 | 13,600 | L-H: <10^-10,^ L-I: <10^-10^ , I-H: <10^-10^ | 91 |
| **GBM** | 153 | 29,143 | 3 | 8,243 | L-H: <10^-10,^ L-I: <10^-10^ , I-H: 0.001 | 98 |
| **HNSC** | 530 | 33,316 | 2 | 21,978 | L-H: <10^-10,^ L-I: <10^-10^ , I-H: <10^-10^ | 98 |
| **KIRC** | 320 | 24,690 | 3 | 8,557 | L-H: <10^-10,^ L-I: <10^-10^ , I-H: <10^-10^ | 91 |
| **KIRP** | 276 | 23,040 | 2 | 4,552 | L-H: <10^-10,^ L-I: <10^-10^ , I-H: <10^-10^ | 99 |
| **LAML** | 140 | 25,710 | 2 | 3,104 | L-H: <10^-10,^ L-I: <10^-10^ , I-H: <10^-10^ | 89 |
| **LGG** | 530 | 21,620 | 2 | 13,642 | L-H: <10^-10,^ L-I: <10^-10^ , I-H: <10^-10^ | 98 |
| **LIHC** | 379 | 30,705 | 3 | 20,521 | L-H: <10^-10,^ L-I: <10^-10^ , I-H: <10^-10^ | 86 |
| **LUAD** | 460 | 28,186 | 3 | 16,628 | L-H: <10^-10,^ L-I: <10^-10^ , I-H: <10^-10^ | 89 |
| **LUSC** | 370 | 33,143 | 3 | 22,250 | L-H: <10^-10,^ L-I: <10^-10^ , I-H: <10^-10^ | 84 |
| **MESO** | 87 | 27,742 | 3 | 586 | L-H: <10^-10,^ L-I: <10^-10^ , I-H: <10^-10^ | 76 |
| **PAAD** | 185 | 26,820 | 3 | 8,936 | L-H: <10^-10,^ L-I: <10^-10^ , I-H: <10^-10^ | 89 |
| **PCPG** | 152 | 37,877 | 3 | 18,920 | L-H: <10^-10,^ L-I: <10^-10^ , I-H: <10^-10^ | 95 |
| **PRAD** | 499 | 20,587 | 2 | 11,861 | L-H: <10^-10,^ L-I: <10^-10^ , I-H: <10^-10^ | 72 |
| **READ** | 99 | 25,888 | 3 | 2,182 | L-H: <10^-10,^ L-I: <10^-10^ , I-H: <10^-10^ | 78 |
| **SARC** | 265 | 31,129 | 2 | 1,275 | L-H: <10^-10,^ L-I: <10^-10^ , I-H: <10^-10^ | 73 |
| **SKCM** | 473 | 31,938 | 3 | 23,973 | L-H: <10^-10,^ L-I: <10^-10^ , I-H: <10^-10^ | 93 |
| **STAD** | 395 | 39,004 | 2 | 20,308 | L-H: <10^-10,^ L-I: <10^-10^ , I-H: <10^-10^ | 99 |
| **THCA** | 515 | 19,628 | 2 | 3,137 | L-H: <10^-10,^ L-I: <10^-10^ , I-H: <10^-10^ | 95 |
| **THYM** | 124 | 23,784 | 2 | 8,282 | L-H: <10^-10,^ L-I: <10^-10^ , I-H: <10^-10^ | 91 |
| **UCEC** | 432 | 24,400 | 3 | 13,851 | L-H: <10^-10,^ L-I: <10^-10^ , I-H: <10^-10^ | 89 |
| **Average (IQR)** | **325 [161-453]** | **28,218 [24,473-31,734]** | **/** | **11,914 [5,211-19,213]** | **/** | **90 [87-95]** |

Supplemental Table 3: Difference between low and high-methylation clusters and high-methylation cluster average beta-value for spectral clustering with ***k*** equal to 2 or 3. The differences are indicated as (*k* = 2, *k* = 3).

| **Cancer** | $\Delta\left( low,high \right)$ for $k=2$, $\Delta\left( low,high \right)$  for $k=3$ | **Average high group** | **Cancer** | $\Delta\left( low,high \right)$ for $k=2$, $\Delta\left( low,high \right)$  for $k=3$ | **Average high group** |
| --- | --- | --- | --- | --- | --- |
| **ACC** | 0.31, 0.42 | 0.56, 0.65 | **LUAD** | 0.14, 0.26 | 0.59, 0.66 |
| **BLCA** | 0.17, 0.17 | 0.63, 0.61 | **LUSC** | 0.14, 0.25 | 0.72, 0.73 |
| **BRCA** | 0.09, 0.11 | 0.56, 0.61 | **MESO** | 0.28, 0.37 | 0.56, 0.59 |
| **CESC** | 0.24, 0.23 | 0.68, 0.66 | **PAAD** | 0.13, 0.19 | 0.54, 0.59 |
| **COAD** | 0.26, 0.32 | 0.57, 0.62 | **PCPG** | 0.27, 0.28 | 0.57, 0.66 |
| **ESCA** | 0.13, 0.17 | 0.53, 0.56 | **PRAD** | 0.13, 0.11 | 0.55, 0.56 |
| **GBM** | 0.17, 0.20 | 0.71, 0.68 | **READ** | 0.24, 0.31 | 0.80, 0.80 |
| **HNSC** | 0.25, 0.20 | 0.64, 0.62 | **SARC** | 0.50, 0.41 | 0.77, 0.79 |
| **KIRC** | 0.21, 0.23 | 0.59, 0.50 | **SKCM** | 0.18, 0.28 | 0.64, 0.68 |
| **KIRP** | 0.57, 0.43 | 0.71, 0.66 | **STAD** | 0.35, 0.33 | 0.62, 0.64 |
| **LAML** | 0.40, 0.28 | 0.70, 0.54 | **THCA** | 0.28, 0.23 | 0.60, 0.56 |
| **LIHC** | 0.22, 0.31 | 0.68, 0.69 | **THYM** | 0.13, 0.12 | 0.53, 0.60 |
| **LGG** | 0.20, 0.21 | 0.53, 0.54 | **UCEC** | 0.14, 0.16 | 0.57, 0.61 |

Supplemental Table 4: Significantly associated clinical variables with cluster membership for 26 cancer types in TCGA. Associations are computed with a Kruskal Wallis test for continuous variables and chi-square for categorical variables (when assumptions are met). *p*-values are Bonferroni corrected.

| **Cancer** | **Clinical variable** | **Categories** | **p** | **Cancer** | **Clinical variable** | **Categories** | **p** |
| --- | --- | --- | --- | --- | --- | --- | --- |
| **ACC** | Days to death | Numerical | 0.0001 | **KIRC** | Year of diagnosis | Numerical | 7x10^-5^ |
| **BLCA** | Diagnosis subtype | Papillary; Non-papillary | 3x10^-15^ | **KIRC** | Days to/year of death | Numerical | 1x10^-6^ |
| **BLCA** | Pathologic diagnosis method | TURBT; Endoscopic Biopsy; Other | 4x10^-7^ | **KIRC** | Longest dimension | Numerical | 2x10^-5^ |
| **BLCA** | Lymph node examined count | Numerical | 0.0001 | **KIRP** | Age at diagnosis | Numerical | 0.0003 |
| **BLCA** | Number of lymphnodes positive by he | Numerical | 7x10^-7^ | **KIRP** | Lymph nodes examined count | Numerical | 9x10^-8^ |
| **BLCA** | Primary lymph node presentation assessment | Numerical | 2x10^-9^ | **KIRP** | Number of lymph nodes positive | Numerical | 3x10^-8^ |
| **BLCA** | Year of birth | Numerical | 7x10^-5^ | **KIRP** | Days to birth | Numerical | 0.0003 |
| **BRCA** | Age at diagnosis | Numerical | 0.0004 | **KIRP** | Days to death | Numerical | 0.0001 |
| **CESC** | Lymph node examined count | Numerical | 0.0002 | **KIRP** | Year of death | Numerical | 0.0001 |
| **ESCA** | Age at diagnosis | Numerical | 9x10^-6^ | **KIRP** | Days to last follow up | Numerical | 2x10^-5^ |
| **ESCA** | Reflux history | Yes; No | 6x10^-5^ | **LGG** | Age at diagnosis | Numerical | 1x10^-11^ |
| **ESCA** | Tissue collection indicator | Yes; No | 6x10^-10^ | **LGG** | Follow up case report form subm. reason | Scheduled Follow-up submission; Additional new tumor event | 0.0002 |
| **ESCA** | Weight/BMI | Numerical | 3x10^-12^ | **LGG** | Neoplasm histologic grade | G2; G3 | 1x10^-7^ |
| **ESCA** | Days/years to birth | Numerical | 8x10^-8^ | **LGG** | New tumor event after init. treatment | Yes; No | 6x10^-6^ |
| **ESCA** | Days to collection | Numerical | 6x10^-6^ | **LGG** | Days to/year of birth | Numerical | 1x10^-11^ |
| **GBM** | Age at diagnosis | Numerical | 0.0003 | **LGG** | Days to/year of death | Numerical | 1x10^-7^ |
| **GBM** | Days to birth | Numerical | 0.0003 | **LGG** | Morphology | 9401/3; 9382/3; 9450/3; 9400/3; 9451/3 | 4x10^-8^ |
| **HNSC** | Age at diagnosis | Numerical | 0.0003 | **LGG** | Primary diagnosis | Astrocytoma, anaplastic or NOS; Oligodendroglioma, anaplastic or NOS; Mixed glioma | 3x10^-8^ |
| **HNSC** | Lymph node examined count | Numerical | 1x10^-6^ | **LIHC** | Age at diagnosis | Numerical | 1x10^-8^ |
| **HNSC** | Lymphnode neck dissection | Yes; No | 3x10^-9^ | **LIHC** | Days to/year of birth | Numerical | 6x10^-8^ |
| **HNSC** | Number of positive lymphnodes by he/ihc | Numerical | 3x10^-5^ | **READ** | Weight | Numerical | 0.0003 |
| **HNSC** | Postoperative rx tx | Yes; No |  | **THCA** | Number of lymphnodes positive he | Numerical | 0.0004 |
| **HNSC** | Primary lymph node assessment | Yes; No | 4x10^-6^ | **UCEC** | Age at diagnosis | Numerical | 7x10^-7^ |
| **HNSC** | Vital status | Alive; Dead | 3x10^-5^ | **UCEC** | Neoplasm cancer status | Tumor free; with tumor | 2x10^-5^ |
| **HNSC** | Days to birth/year of birth | Numerical | 1x10^-6^ | **UCEC** | Postoperative rx tx | Yes; No | 2x10^-12^ |
| **HNSC** | Days to death/year of death | Numerical | 0.0003 | **UCEC** | Weight | Numerical | 0.0002 |
| **HNSC** | Days to last follow up | Numerical | 7x10^-6^ | **UCEC** | Days to/year of birth | Numerical | 1x10^-6^ |
| **KIRC** | Days to new tumor event after initial treatment | Numerical | 4x10^-5^ |  |  |  |  |

Supplemental Table 5: Methylation Genes Mutation Enrichment (MGME) analysis of COAD genes. The empirical *p*-value is computed through a repeated (500-fold) draw of similar genes to the gene of interest and the computation of the associated MGME (Supplemental Appendix). *p*-values are FDR-corrected (Benjamini-Hochberg) and significant mutations (p<0.1) are indicated by (*). Genes whose mutation status is associated with CIMP after the correction for the total mutation frequency is highlighted in bold.

| **Gene** | **p** | **Gene** | **p** | **Gene** | **p** | **Gene** | **p** |
| --- | --- | --- | --- | --- | --- | --- | --- |
| **ASH1L*** | <0.01 | PRDM10 | 0.40 | TET2 | 0.40 | KMD5C | 0.40 |
| **EHMT1*** | 0.09 | **PRDM13*** | <0.01 | TET3 | 0.44 | KDM6A | 0.40 |
| EHMT2 | 0.40 | **PRDM14*** | 0.06 | MBD1 | 0.40 | KDM6B | 0.44 |
| KMT2A | 0.29 | PRDM15 | 0.40 | **APOBEC1*** | 0.07 | PHF2 | 0.40 |
| **KMT2B*** | 0.01 | PRDM16 | 0.13 | CTCF | 0.40 | **BRAF*** | <0.01 |
| KMT2C | 0.24 | **SETD1A*** | <0.01 | KDM2B | 0.40 | **KRAS*** | 0.05 |
| KMT2D | 0.44 | **SETD1B*** | 0.05 | KDM3B | 0.40 |  |  |
| **MECOM*** | 0.01 | SETD2 | 0.13 | **KDM4A*** | 0.01 |  |  |
| NSD1 | 0.40 | **BAZ2A*** | 0.01 | **KDM4B*** | 0.07 |  |  |
| **PRDM1*** | 0.02 | **DNMT1*** | 0.01 | KDM4C | 0.17 |  |  |
| PRDM2 | 0.40 | UHRF1BP1L | 0.40 | KDM5A | 0.40 |  |  |
| PRDM9 | 0.45 | TET1 | 0.40 | KDM5B | 0.40 |  |  |

Supplemental Table 6: Cancer types presenting ***IDH1*** and ***SETD2*** mutations significantly correlated with the CIMP score. We rank patients according to their CIMP score and compute the point-biserial correlation between the mutation status and the score for *IDH1* and *SETD2* mutations. We report the number (#) of patients presenting a mutation (% of the total population), the correlation coefficient r and the FDR Benjamini-Hochberg corrected *q* value. Significant *q* values (*q*<0.1) are indicated by (*).

| **Cancer** | **# (%) of patients presenting mutation in *IDH1*, *SETD2*** | **r, q for *IDH1*** | **r, q for *SETD2*** | **Cancer** | **# of patients presenting mutation in *IDH1*, *SETD2*** | **r, q for *IDH1*** | **r, q for *SETD2*** |
| --- | --- | --- | --- | --- | --- | --- | --- |
| **COAD** | 5 (2%), 21 (7%) | 0.2, 0.009* | 0.4, 1x10^-9^* | **LUAD** | 6 (1%), 32 (7%) | 0.1, 0.01* | 0.01, 0.38 |
| **GBM** | 7 (5%), 7 (5%) | 0.2, 0.07* | -0.3, 0.002* | **MESO** | 0 (0%), 8 (9%) | NA, NA | 0.3, 0.08* |
| **KIRC** | 0 (0%), 39 (12%) | NA, NA | 0.3, 4x10^-6^* | **PRAD** | 5 (1%), 6 (1%) | 0.1, 0.03* | 0.05, 0.26 |
| **KIRP** | 0 (0%), 17 (6%) | NA, NA | 0.1, 0.1* | **SKCM** | 23 (5%), 26 (6%) | 0.1, 0.01* | 0.04, 0.39 |
| **LAML** | 16 (11%), 1 (<1%) | 0.4, 1x10^-6^* | -0.07, 0.41 | **UCEC** | 18 (4%), 61 (14%) | 0.1, 0.03* | 0.2, 2x10^-5^* |
| **LGG** | 397 (75%), 10 (2%) | 0.7, 2x10^-78^* | -0.1, 0.09* |  |  |  |  |

Supplemental Table 7: Statistical testing of associations between CIMP and overall patients’ survival, univariate analysis. We report log-rank test (LR) associated *p*-values with Benjamini-Hochberg False Discovery Rate (FDR) correction. The LR FDR values are indicated in the following order: low versus intermediate, low versus high, intermediate versus high. Significant values are italicized. Differences that have not yet been described in the literature are indicated by an asterisk (*).

| **Cancer** | **LR FDR** | **Cancer** | **LR FDR** |
| --- | --- | --- | --- |
| **ACC** | *0.04,* *2x10^-5^,* 0.34 | **LUAD** | 0.91, 0.36, 0.32 |
| **CESC** | 0.40 | **LUSC** | 0.22, 0.53, 0.32 |
| **COAD** | 0.84, 0.79, 0.88 | **MESO** | 0.49, *0.003, 2x10^-5^* |
| **GBM** | 0.92, *0.04, 0.04* | **PCPG** | 0.81, 0.69, 0.80 |
| **HNSC** | 0.38, *0.05*, 0.005** | **READ** | 0.91, 0.80, 0.95 |
| **KIRC** | 0.41, *0.04, 5x10^-5^* | **SARC** | 0.91 |
| **KIRP** | *5x10^-18^* | **SKCM** | 0.05, 0.90, *0.04** |
| **LAML** | 0.54 | **STAD** | 0.60 |
| **LGG** | *1x10^-24^* | **THCA** | 0.05 |
| **LIHC** | 0.41, 0.37, 0.95 |  |  |

Supplemental Table 8: Statistical testing of associations between CIMP and overall patients’ survival, multivariate analysis with Cox regression. We report Cox regression model *p*-value associated with the methylation group, Cox regression model Hazard Ratio (95% Confidence Interval) and median of the predicted median survival time in low-methylation versus high-methylation group (NA if the Kaplan Meyer estimate does not cross 0.5), Cox regression model *p*-value associated with stage, age and gender when applicable and the Cox regression model Hazard Ratio for said covariates. The Cox *p*-values for the methylation groups are indicated in the following order: low versus intermediate, low versus high; the Cox *p*-values for covariates are indicated in the order of indicated covariates. Significant values are italicized. Differences that have not yet been described are indicated by an asterisk (*).

| **Cancer** | **Cox *p* methylation group (low *vs*. intermediate, low *vs*. high)** | **Cox methylation group HR (95% CI)** | **Median survival time low versus high (d)** | **Covariates** | **Cox *p* covariates** | **Cox covariate HR** |
| --- | --- | --- | --- | --- | --- | --- |
| **ACC** | 0.22, *0.0009* | 1.7 [0.7-4.0],*4.4 [1.8-10.6]* | *NA vs 1197* | Stage, age, gender, | <*10^-4^*, 0.22, 0.98 | *2.2*, 1.3, 1.0 |
| **CESC** | 0.37 | 0.8 [0.5-1.4] | 3,046 vs NA | Stage, age | *0.02*, 0.50 | *1.3*, 1.1 |
| **COAD** | 0.78, 0.71 | 1.1 [0.7-1.7], 1.1 [0.6-2.2] | NA vs NA | Stage, age, gender | <*10^-4^*, 0.78, 0.71 | *1.7*, 1.3, 0.8 |
| **GBM** | 0.31, 0.15 | 0.8 [0.5-1.2], 0.5 [0.2-1.3] | 399 vs 1,448 | Age, gender | <*10^-4^*, *0.009* | *1.6, 0.6* |
| **HNSC** | 0.13, *0.03** | 1.3 [0.9-1.9], *0.5 [0.2-0.9]* | 2,002 vs 4,760 | Stage, age, gender | <*10^-4^*, *0.02*, 0.18 | *1.4, 1.2,* 1.2 |
| **KIRC** | 0.76, 0.36 | 0.9 [0.5-1.6],1.3 [0.7-2.4] | NA vs 1,091 | Stage, age, gender | <*10^-4^*, *0.002*, 0.91 | *1.7, 1.4*, 1.0 |
| **KIRP** | *0.0002* | *6.8 [2.5 - 18.5]* | *NA vs 492* | Stage, age, gender | *0.0002,* 0.43, 0.38 | *1.6,* 1.1, 1.3 |
| **LAML** | 0.62 | 1.2 [0.6-2.3] | 915 vs 517 | Age, gender | *0.0003,* 0.34 | *1.7,* 1.3 |
| **LGG** | *2x10^-12^* | *0.3 [0.2-0.4]* | *814 vs 2,907* | Age, gender | <*10^-4^*, 0.45 | *1.6,* 0.9 |
| **LIHC** | 0.46, 0.68 | 0.9 [0.6-1.3], 0.9 [0.6-1.4] | 2,131 vs 2,532 | Stage, age, gender | *0.0003,* 0.26, 0.91 | *1.4*, 1.1, 1.0 |
| **LUAD** | 0.93, 0.14 | 1.0 [0.6-1.5], 0.6 [0.3-1.2] | 1,622 vs 2,620 | Stage, age, gender | <*10^-4^*, 0.37, 0.44 | *1.6*, 1.1, 1.1 |
| **LUSC** | 0.13, 0.65 | 1.4 [0.9-2.1], 1.1 [0.7-1.7] | 1,984 vs 1,856 | Stage, age, gender | *0.007*, 0.44, 0.90 | *1.3*, 1.1, 1.0 |
| **MESO** | 0.23*, 0.002* | 0.7 [0.4-1.2], *3.5 [1.6-7.7]* | 709 vs 243 | Stage, age, gender | 0.70, 0.55, 0.89 | 1.1, 1.1, 1.0 |
| **PCPG** | 0.95, 0.79 | 1.0 [0.3 - 2.7], 0.8 [0.1-4.8] | NA vs NA | Age, gender | 0.92, 0.72 | 1.0, 0.8 |
| **READ** | 0.41, 0.85 | 1.0 [0.3-3.1],1.1 [0.4 - 3.2] | NA vs NA | Stage, age, gender | 0.38, *0.02*, 0.50 | 1.2, *1.8,* 1.4 |
| **SARC** | 0.67 | 0.8 [0.3-2.1] | NA vs 1,970 | Age, gender | *0.03*, 0.72 | *1.3*, 0.9 |
| **SKCM** | 0.06, 0.53 | 1.3 [1.0-1.8],0.8 [0.5-1.3] | 2,470 vs 3,139 | Stage, age, gender | *0.0002,* <*10^-4^,* 0.95 | *1.3, 1.3*, 1.0 |
| **STAD** | 0.44 | 0.8 [0.5-1.4] | 1,043 vs 1,686 | Stage, age, gender | *0.0001, 0.02*, 0.38 | *1.4, 1.2*, 0.9 |
| **THCA** | 0.35 | 1.8 [0.5-5.9] | NA vs NA | Stage, age, gender | 0.17, *0.001*, 0.55 | 1.2, *1.5*, 0.8 |

**Supplemental Table 9: Probes used for CIMP and non-CIMP classification of 19 cancer types and their associated adjusted balanced accuracy (ABAC) and test accuracy (ACC).** We train a Logistic Regression (90%/10% training/test set split, balanced class weights, 5-fold cross-validation) on k probes (k from 1 to 5) and indicate the average ABAC on the 5-fold cross-validation for the training set as well as the ABAC and accuracy score for the held-out test set.

| **Cancer** | **ABAC (5-cv)** | ***N*** | **Probes selected** | **ABAC/ACC test** | **Cancer** | **ABAC**  **(5-cv)** | ***N*** | **Probes selected** | **ABAC/ACC test** |
| --- | --- | --- | --- | --- | --- | --- | --- | --- | --- |
| **ACC** | 1.000 | 4 | cg02916816cg25902889cg00021532cg00044995 | 1.000/1.000 | **LUAD** | 0.988 | 5 | cg17357161cg10478435cg01453694cg03603211  cg04164184 | 0.738/0.913 |
| **CESC** | 1.000 | 4 | cg04084892cg13759674cg18144285cg01637551 | 0.9000/0.968 | **LUSC** | 0.953 | 5 | cg06471905cg04464062cg07598755cg17360781cg22389121 | 0.784/0.811 |
| **COAD** | 0.99 | 5 | cg12678562  cg17264240  cg22934970  cg14962509  cg14208573 | 0.9000/0.967 | **MESO** | 1.000 | 3 | cg21375294  cg08580187cg04098339 | 1.000/1.000 |
| **GBM** | 1.000 | 1 | cg02486545 | 1.000/1.000 | **PCPG** | 1.000 | 1 | cg01888566 | 1.000/1.000 |
| **HNSC** | 0.984 | 4 | cg07836815cg06017028cg17218813cg2202279 | 0.929/0.981 | **READ** | 0.938 | 2 | cg03392960  cg00106923 | 1.000/1.000 |
| **KIRC** | 0.979 | 5 | cg24496475cg16683160cg23929344cg17406383cg00049033 | 0.833/0.875 | **SARC** | 1.000 | 2 | cg10496150 cg02980127 | 1.000/1.000 |
| **KIRP** | 1.000 | 1 | cg00081574 | 1.000/1.000 | **SKCM** | 0.978 | 5 | cg10530164cg22880757cg07256473cg18933494cg19115272 | 0.752/0.875 |
| **LAML** | 0.996 | 1 | cg13652493 | 1.000/1.000 | **STAD** | 1.000 | 1 | cg16412592 | 1.000/1.000 |
| **LGG** | 0.999 | 2 | cg05866411cg00004072 | 0.989/0.981 | **THCA** | 0.999 | 4 | cg03571320cg17590003cg04013090cg00997280 | 1.000/1.000 |
| **LIHC** | 0.976 | 5 | cg13397345cg00351735cg06076512cg16201146cg02737307 | 0.962/0.973 | **Avg [IQR]** | **0.989 [0.981-1.000]** | **/** | **/** | **0.936 [0.900-1.000] / 0.965 [0.967-1.000]** |

Supplemental Table 10: EASE score (gene set enrichment analysis) of downstream events. We select genes that are both differentially expressed and with associated hypermethylated probes in the high-methylation group. We use the EASE score, a modified version of Fisher’s exact test - technique developed in DAVID (<https://david.ncifcrf.gov/>) - to compute the gene set enrichment analysis, using the updated gene sets obtained from the Broad Institute website (<https://www.gsea-msigdb.org/gsea/msigdb/>). The set of enriched pathways of LGG and LIHC are only partially displayed; the full list is available on Github.

| **Cancer** | **EASE score (FDR *q*-value)** |
| --- | --- |
| ACC | Pattern specification process (10^-6^), regionalization (5x10^-6^), neuron differentiation (1.4x10^-5^), neurogenesis (9.5x10^-5^), central nervous system development (1.2x10^-4^), anterior/posterior pattern specification (2.9x10^-4^), pos. Reg. of nucleobase content compound metab. Process (4.5x10^-4^), pos. Reg. biosynthetic process (8x10^-4^), embryo development (1.9x10^-3^), head development (0.003), cell fate commitment (0.03), skeletal system development (0.04) |
| CESC | homophilic/cell cell adhesion via plasma membrane adhesion molecules (9x10^-10^), animal organ morphogenesis (7.2x10^-5^), pattern specification process (1.4x10^-4^), skeletal system development (1.6x10^-4^), cell-cell adhesion (2.7x10^-4^), biological adhesion (3x10^-4^), embryonic organ development (5.5x10^-4^), positive reg of biosynthetic process (7.2x10^-4^), central nervous system dev (1.9x10^-3^), embryo dev (0.002), head dev (0.006), regionalization (0.007), embryonic organ morphogenesis (0.008), embryonic morphogenesis (0.009), sensory organ dev (0.02), cell cell signaling (0.04), pos reg of nucleobase containing metabolic process (0.05) |
| COAD | Central nervous system dev (10^-6^), head dev (1.6x10^-5^), embryo dev (2.7x10^-4^), neural precursor cell proliferation (0.002), animal organ morphogenesis (0.002), neuron differentiation (0.002), neurogenesis (0.003), sensory organ dev (0.01), reg of neural precursor cell proliferation (0.02), embryonic morphogenesis (0.02), cell cell signaling (0.02), cell fate commitment (0.02), regionalization (0.02), synapse organization (0.03), pattern specification process (0.04) |
| GBM | None |
| HNSC | Nervous system process (1x10^-6^), transmembrane transport (1.7x10^-5^), central nervous system development (2.4x10^-5^), neuron differentiation (5x10^-5^), neurogenesis (1.8x10^-4^), behavior (1.8x10^-4^), neuron development (1.4x10^-3^), sensory organ dev (0.003), cation transport (0.003), inorganic ion transmembrane transport (0.003), cell cell signaling (0.004), animal organ morphogenesis (0.009), sensory perception (0.01), cell fate specification (0.02), cation transmembrane transport (0.02), ear dev (0.04), sensory organ morphogenesis (0.04) |
| KIRC | Reg of hormone levels (0.01), phenol compound containing metabolic process (0.02), animal organ morphogeness (0.02), cell cell signaling (0.04), cell junction organization (0.05) |
| KIRP | None |
| LAML | None |
| LGG | Embryo development (<10^-30^), embryonic organ dev (<10^-30^), embryonic morphogenesis (<10^-30^), regionalization (<10^-30^), pattern specification process (8.5x10^-13^), animal organ morphogenesis (8.5x10^-13^), homophilic/cell cell adhesion via plasma membrane adhesion molecules (1.4x10^-11^), cell cell adhesion (1x10^-10^), cell fate commitment (3.5x10^-10^), biological adhesion (1.5x10^-9^), embryonic organ morphogenesis (2.5x10^-8^), embryo dev ending in birth or egg hatching (3.8x10^-8^), epithelium dev (1.5x10^-7^), anterior posterior pattern specification (5x10^-7^), proximal distal pattern formation (7.4x10^-7^), skeletal system dev (9.5x10^-7^), sensory organ dev (2.6x10^-6^), pos reg of cell differentiation (8x10^-6^), pos reg of transcription by RNAPII (2.2x10^-5^), ... |
| LIHC | Animal organ morphogenesis (<10^-30^), homophilic/cell cell adhesion via plasma membrane adhesion molecules (<10^-30^), neuron differentiation (<10^-30^), neurogenesis (<10^-30^), cell cell signaling (1.7x10^-12^), sensory organ dev (8.5x10^-12^), neuron dev (5.1x10^-11^), embryonic morphogenesis (7.3x10^-11^), cell part morphogenesis (8.8x10^-11^), cellular component morphogenesis (1x10^-10^), embryonic organ morphogenesis (1.9x10^-10^), biological adhesion (2.5x10^-10^), embryo dev (8.9x10^-10^), embryonic organ dev (1.2x10^-9^), skeletal system dev (5.3x10^-9^), cation transmembrane transport (6.5x10^-9^), regionalization (1.2x10^-8^), cell morphogenesis involved in neuron differentiation (1.2x10^-8^), pattern specification process (1.6x10^-8^), cell morphogenesis (1.7x10^-8^), ... |
| LUAD | Neuron differentiation (1.5x10^-7^), neurogenesis (6.4x10^-7^), central nervous system development (1.2x10^-6^), cell cell signaling (1.7x10^-6^), head dev (5.5x10^-6^), pattern specification process (3.1x10^-4^), regionalization (6.2x10^-4^), signal release (1.3x10^-3^), neuron dev (1.4x10^-3^), embryonic morphogenesis (2.3x10^-3^), population proliferation (3.3x10^-3^), cell part morphogenesis (0.005), animal organ morphogenesis (0.006), central nervous system neuron differentiation (0.009), hindbrain dev (0.01), embryo dev (0.01), reg of hormone levels (0.02), cellular component morphogenesis (0.02), neuroactive ligand receptor interaction (0.04), synaptic signaling (0.04) |
| LUSC | None |
| MESO | None |
| PCPG | None |
| READ | None |
| SARC | None |
| SKCM | None |
| STAD | Regionalization (1.3x10^-9^), homophilic/cell cell adhesion via plasma membrane adhesion molecules (2.5x10^-9^), pattern specification process (3.4x10^-9^), embryo development (8.7x10^-6^), cation transport (2.5x10^-4^), animal organ morphogenesis (2.9x10^-4^), anterior posterior pattern specification (5.8x10^-4^), cell population proliferation (1.3x10^-3^), embryonic organ development (1.4x10^-3^), pos reg of transcription by RNAPII (1.4x10^-3^), epithelial cell differentiation (1.7x10^-3^), epithelium dev (0.002), import into cell (0.002), potassium ion transport (0.003), cell fate specification (0.003), cell cell adhesion (0.004), cation transmembrane transport (0.004), embryonic morphogenesis (0.005), appendage dev (0.006), neurogenesis (0.009), pos reg of biosynthetic process (0.01), embryonic organ morphogenesis (0.02), reg of transmembrane transport (0.02), neuron differentiation (0.02), reg of hormone levels (0.02), pos reg of nucleobase containing compound metabolic process (0.03), cell cell signaling (0.03), inorganic ion transmembrane transport (0.03), reproductive system dev (0.04), biological adhesion (0.04) |
| THCA | None |

Supplemental Table 11: Putative drivers of the high methylation group among mutations and gene expression changes for 7 CIMP-negative cancer types. The putative mutations were extracted from the mutation analysis with the Random Forest method. Differential gene expression (Diff. GEX) was computed through DESeq2 between high and low-methylation groups for genes involved in DNA and histone methylation. Mutations are indicated as (low-methylation group %/high-methylation group %), gene expression is indicated as Fold Change, FC, between high and low-methylation groups. (FC>1 corresponds to overexpression in the high-methylation group). Only significant mutations (Fisher exact test *p*<0.05) with a difference >10% between the low and high-methylation groups were reported. Mutations that did not pass the 0.1 threshold on q-value are indicated by non-significant (NS).

| **Cancer** | **(De)Methylation**  **mutation** | **Diff. GEX** | **Non-meth. mutation** | **Cancer** | **(De)Methylation**  **mutation** | **Diff. GEX** | **Non-meth. mutation** |
| --- | --- | --- | --- | --- | --- | --- | --- |
| **BLCA** | KMT2C (27%/16%) (NS), KDM6A (38%/19%) | **-** | FGFR3 (12%/5%) (NS), TP53 (63%/51%) (NS), STAG2 (13%/9%) (NS), RB1 (16%/23%) (NS), ERBB2 (7%/16%) (NS) | **PRAD** | - | **-** | TTN (3%/12%), SPOP (6%/12%) (NS), FOXA1 (0%/7%), TP53 (4%/13%) |
| **BRCA** | **-** | **-** | TP53 (20%/22%) (NS), PIK3CA (41%/41%) (NS), CDH1 (14%/22%), GATA3 (12%/16%) (NS) | **THYM** | **-** | - |  |
| **ESCA** | - | - | LAMA1 (2%/26%), NFE2L2 (2%/0%) (NS) | **UCEC** | ASH1L (1%/26%), DOT1L (1%/13%), EHMT2 (2%/15%), EZH1 (1%/12%), EZH2 (0%17%), KMT2A (3%/24%), KMT2B (1%/55%), KMT2C (4%/30%), KMT2D (6%/52%), NSD1 (0%/33%), PRDM10 (0%/14%), PRDM11 (0%/12%), PRDM15 (2%/15%), PRDM2 (1%/14%), PRDM5 (1%/14%), PRDM9 (3%/16%), SETD1B (4%/34%), SETD2 (1%/29%), SETDB1 (1%/17%), SUV39H1 (1%/12%), WHSC1 (1%/15%), WHSC1L1 (1%/23%), JMJD1C (3%/22%), KDM2B (1%/15%), KDM3B (3%/25%), KDM4A (0%12%), KDM4B (2%/14%), KDM4C (0%/11%), KDM5A (1%/17%),KDM5C (3%/20%), KDM6B (1%/22%), PHF2 (1%/14%), PHF8 (1%/15%), TET1 (1%/18%), TET2 (1%/18%), TET3 (1%/22%), MDB1 (1%/12%), MBD4 (0%/10%), CTCF (2%/43%), IDH1 (0%/11%), IDH2 (0%/13%), BAZ2A (1%/13%), CTCFL (1%/12%), DNMT1 (2%/16%), DNMT3A (0%/15%), DNMT3B (1%/15%), UHRF1BP1 (2%/14%), UHRF1BP1L (2%/12%), ARID1A (4%/76%) | - | PTEN (6%/82%), TP53 (86%/27%)  CTNNB1 (0%/23%), KRAS (2%/30%), PPP2R1A (36%/16%) |
| **PAAD** | **-** | - | KRAS (10%/97%), TP53 (20%/71%), SMAD4 (7%/44%), RNF43 (0%/26%), TTN (3%/29%), CDKN2A (7%/26%) |  | - | **-** | - |

Supplemental Table 12: Statistical testing of associations between the high methylation group and overall patients’ survival for CIMP-negative cancer types. We state log-rank test (LR) associated *p*-values with Benjamini-Hochberg False Discovery Rate (FDR) correction, Cox regression model *p*-value associated with group, Cox regression model Hazard Ratio (95% Confidence Interval) and median of the predicted median survival time in low-methylation versus high-methylation group (NA if the Kaplan Meyer estimate does not cross 0.5). The LR FDR values are indicated in the following order: low versus intermediate, low versus high, intermediate versus high. The Cox *p*-values are indicated in the following order: low versus intermediate, low versus high. Significant values are italicized.

| **Cancer** | **LR FDR** | **Cox p** | **Cox group HR (95% CI)** | **Median survival time low versus high (d)** | **Cancer** | **LR FDR** | **Cox p** | **Cox group HR (95% CI)** | **Median survival time low versus high (d)** |
| --- | --- | --- | --- | --- | --- | --- | --- | --- | --- |
| **BLCA** | 0.13, 0.94, 0.08 | 0.37, 0.71 | 0.8 [0.5-1.3],1.1 [0.8-1.5] | 1,008 vs 1,004 | **PRAD** | 0.93 | 0.91 | 1.0 [0.4-2.2] | NA vs NA |
| **BRCA** | 1.0, 0.89, 0.88 | 0.77, 0.73 | 1.1 [0.7-1.6], 0.9 [0.7-1.3] | 3,959 vs 3,959 | **THYM** | 0.70 | 0.77 | 0.9 [0.3-2.5] | NA vs NA |
| **ESCA** | 0.98, 0.93,, 0.88 | 0.45, 0.90 | 1.3 [0.7-2.2], 1.0 [0.6-1.8] | 730 vs 1,263 | **UCEC** | *0.02,* 0.13, 0.89 | 0.24, 0.55 | 0.8 [0.5-1.2], 0.9 [0.5-1.4] | 3,112 vs NA |
| **PAAD** | 0.15, 0.46, 1.0 | 0.22, 0.41 | 1.4 [0.8-2.2], 1.3 [0.7-2.4] | 738 vs 598 |  |  |  |  |  |

###

### Supplemental appendix

#### List of studied genes

Methylation associated genes [[109–112]](https://paperpile.com/c/NzlxcQ/Xj9zn+99vW8+e3jSF+jkxU1): BAZ2A, CTCFL, DMAP1, DNMT1, DNMT3A, DNMT3B, UHRF1, UHRF1BP1, UHRF1BP1L

Demethylation associated genes [[109,113]](https://paperpile.com/c/NzlxcQ/Xj9zn+MjRDz):

TET1, TET2, TET3, TDG, MBD1, MBD2, MBD4, CTCF, IDH1, IDH2

Histone methylation associated genes [[114]](https://paperpile.com/c/NzlxcQ/vCjCI):

H3K4 methylation: SETD1A, SETD1B, KMT2A, KMT2D, KMT2C, KMT2B, SMYD1, SMYD2, SETD7, SETD9, PRDM9

H3K9 methylation: SUV39H1, SUV39H2, EHMT2, EHMT1, SETD1B, PRDM1, PRDM2, MECOM, PRDM4, PRDM5, PRDM6, PRDM7, PRDM8, PRDM9, PRDM10, PRDM11, PRDM12, PRDM13, PRDM14, PRDM15, PRDM16

H3K27: EZH1, EZH2

H3K36: SETD2, NSD1, WHSC1, WHSC1L1, SMYD2, ASH1L, SETD3, SETMAR

H3K79: DOT1L

H4K20: SETD8, SUV420H1, SUV420H2

Histone demethylation associated genes[[114]](https://paperpile.com/c/NzlxcQ/vCjCI):

H3K4: KDM1A, KDM1B, KDM5A, KDM5B, KDM5C, KDM5D

H3K9: KDM3B, KDM3A, KDM4A, KDM4B, KDM4C, KDM4D, KDM4E, PHF8, JMJD1C

H3K27: KDM6A, UTY, KDM6B, JHDM1D, PHF8

H3K36: KDM2A, KDM2B, KDM4A, KDM4B, KDM4C, KDM4D

H4K20: PHF8, PHF2

#### Geo Accession numbers for external normal samples

ACC [[115]](https://paperpile.com/c/NzlxcQ/s6D6G): GSM2060850, GSM2060851, GSM2060852, GSM2060853, GSM2060854, GSM2060855,

LAML [[116]](https://paperpile.com/c/NzlxcQ/kdzH2): GSM796661, GSM796662, GSM796665, GSM796666, GSM796668, GSM796669, GSM796670, GSM796671, GSM796672, GSM796673, GSM796674, GSM796675, GSM796676, GSM796677

####

### Supplemental Methods

#### Datasets

We used methylation data from the Infinium® HumanMethylation450 BeadChip (450K Illumina) arrays, that interrogates 485,764 cytosine positions of the human genome, out of which 482,421 positions (99.3%) are CpG dinucleotides [[117]](https://paperpile.com/c/xWfVdX/BSFL3). The metric evaluated is the beta-value approximating the percentage of methylated cytosines across cells, which is defined as $\frac{M}{(M+U+a)}$ with $M\geq0,U\geq0$ the methylated and unmethylated signal intensities respectively, and $a\geq0$ the offset [[118]](https://paperpile.com/c/xWfVdX/TMNsZ).

We used the mRNA expression data from the Illumina HiSeq platform in raw read counts for the differential gene expression analysis. To associate genomic variants with CIMP, we used binary non-silent mutation information provided by the Multi-Center Mutation Calling in Multiple Cancers project (MC3) [[119]](https://paperpile.com/c/xWfVdX/Fy3en). We used tumor purities as provided by Consensus Purity Estimation (CPE) [[48]](https://paperpile.com/c/xWfVdX/MiEin) and ESTIMATE scores for cancer types with no CPE information [[120]](https://paperpile.com/c/xWfVdX/YyX5A).

To identify putative driver events such as over- or under-expression of genes involved in DNA or histone methylation or demethylation, we performed differential gene expression analysis between groups using DESeq2 [[121]](https://paperpile.com/c/xWfVdX/DmCip) on raw count data available on the GDC portal.

#### Individual-level methylation deconvolution with abundant subtype approximation

Here we describe the model used for our deconvolution strategy designed to remove the tumor microenvironment component from the beta-values observed for tumor samples. Let $n$ be the number of samples of a cancer type and $m$ the number of probes remaining after the first filtering steps. Let $\boldsymbol{X}\in\mathbb{R}^{n\times m}$ be the matrix of methylation values for all variable probes selected as described above, $\boldsymbol{A}\in\mathbb{R}^{n\times2}$ the proportion matrix such that $A_{i}=(a_{i}^{1},a_{i}^{2})$ with $a_{i}^{1}$ the purity of tumor sample $i$ and $a_{i}^{2}=1-a_{i}^{1}$ the fraction of tumor microenvironment cells in tumor sample $i$, and $\bar{\boldsymbol{S}}\in\mathbb{R}^{2\times m}$ the cell subtype matrix, where $\bar{S^{j}}$ the average DNA methylation beta-values of subtype $j$ ($j\in\{1,2\}$ with $1$ being cancerous tissue and $2$ the tumor microenvironment). We model

$$\boldsymbol{X}\approx\boldsymbol{A}\bar{\boldsymbol{S}}$$

For the TCGA data, the matrix $\boldsymbol{A}$ describing tumor purity is assumed to be known. With known $\boldsymbol{A}$, we can apply the debCAM method [[51]](https://paperpile.com/c/xWfVdX/qqjke), which is equivalent to performing non-negative matrix factorization (NMF) with non-negative least squares (NNLS). We include the constraint that methylation values are sampled from $[0,1]$ by performing the clipping transformation on the elements of $\bar{\boldsymbol{S}}$:

$x=\left\{ x if x\in[0,1] else 1 if x>1 else 0 if x<0 \right\}$.

Once we obtain $\bar{\boldsymbol{S}}$, we represent the individual methylation values using the following model [[52]](https://paperpile.com/c/xWfVdX/WTZ2i):

$$X_{i}=a_{i}\left( \bar{\boldsymbol{S}}+\boldsymbol{\Delta}\boldsymbol{S}_{\boldsymbol{i}} \right),\forall i\in\left\{ 1,...,n \right\}$$

(1)

where $\boldsymbol{\Delta}\boldsymbol{S}_{\boldsymbol{i}}\in\mathbb{R}^{2\times m}$ is the patient $i$ specific subtype DNA methylation variation. We have

$$\left( 1 \right)\Leftrightarrow X_{i} = a_{i}^{1}\left( \bar{S}^{1}+\Delta S_{i}^{1} \right)+ a_{i}^{2}\left( \bar{S}^{2}+\Delta S_{i}^{2} \right).$$

Let us denote $(\bar{S^{1}}+\Delta S_{i}^{1}) = s_{i}^{1}$, where $s_{i}^{1}$ the individual value of DNA methylation for patient $i$ and subtype $1$ (*i.e.*, cancer cell-specific DNA methylation values). We thus get:

$$s_{i}^{1}\simeq\frac{X_{i} - a_{i}^{2}\bar{S}^{2}}{a_{i}^{1}}$$

Using the fact cancerous cells are usually more abundant as compared to the tumor microenvironment cells ($a_{i}^{2}$ is close to zero) and that we expect small variation in the average DNA methylation values in the tumor microenvironment ($\Delta S_{i}^{2}$ is close to zero), we approximate $a_{i}^{2}\Delta S_{i}^{2}\simeq0$ and thus:

$s_{i}^{1}\simeq\frac{X_{i} - a_{i}^{2}\bar{S}^{2}}{a_{i}^{1}}$.

This allows us to compute the full matrix of methylation in malignant cells as follows:

$S^{1}\simeq(X-A^{2}\bar{S}^{2})⊘A^{1}$,

where $⊘$ is the Hadamard division). Once again, we clip the values so that they belong to interval $[0,1]$. We validated the use of this technique first by observing that any gradient present in the UMAP embedding prior to purity correction disappeared after performing the correction (Supplemental Figure 2); second, by running the pipeline on simulated mixed data and observing an average Mean Absolute Error (MAE) of 0.02 between the simulated ground truth values of methylation in cancerous cells and the values reconstructed by our approach (see below).

#### Artificial data simulation for purity correction validation

To computationally confirm the validity of the purity correction, we simulated mixed DNA methylation data. For a single probe $k$, beta-values for cancerous cells and tumor microenvironment (infiltrate) cells were simulated for each patient $i\in\{1:n\}$ with the normal distribution $\beta_{cancer,i}^{k}\sim\mathcal{N}(\mu_{cancer}^{k},\sigma_{cancer}^{k})$ and $\beta_{infiltrate,i}^{k}\sim\mathcal{N}(\mu_{infiltrate}^{k},\sigma_{infiltrate}^{k})$ where $\beta_{cancer,i}^{k}$ and$\beta_{infiltrate,i}^{k}$ were further bounded to$[0,1]$. We sampled the tumor purity values (${purity}_{i}$) for each patient $i$ from experimental purity values observed the LUSC and LUAD cancers; LUSC and LUAD were chosen for their large variability in tumor purity values. We then created the mixed data by combining $\beta_{cancer}$ and $\beta_{infiltrate}$ using ${purity}_{i}$:

$$\beta_{i}={purity}_{i}*\beta_{cancer,i}+(1-{purity}_{i})*\beta_{infiltrate,i}$$

for each patient $i$.

We created an artificial dataset with 1,000 probes, where $\mu_{cancer}^{k},\sigma_{cancer}^{k},\mu_{infiltrate}^{k},\sigma_{infiltrate}^{k}$ were randomly selected from the following values: $\mu_{cancer}^{k}\in[0.1,0.4,0.6,0.9]$, $\mu_{infiltrate}^{k}\in[0,0.25,0.5,0.75,1]$, $\sigma_{cancer}^{k}\in[0.15,0.25,0.4]$ and $\sigma_{infiltrate}^{k}=0.05$. These values were chosen so that the distribution of beta-values for probes corresponded to that observed on 60 random probes of the LUAD or LUSC dataset; $\sigma_{infiltrate}^{k}$ was chosen using the mode SD in the normal adjacent tissue.

In our simulated experiment, the Mean Absolute Error (MEA), *i.e.*, mean absolute difference between the deconvoluted beta-value and true beta-value was 0.02 on average, which significantly improved over the average MAE (0.11) for the original mixed observations and the ground truth.

#### Selection of the number of clusters

To choose the number *k* of DNA methylation clusters, we tested breaking the set of samples of each cancer type into 2 or 3 clusters and compared the distribution of average beta-values as well as the associated average sample silhouette coefficient (SSC). We selected the number of clusters to increase as much as possible the separability of our low- and high-methylation clusters in the following manner:

1. We chose *k* that maximized the difference in the average beta-value between the low and high-methylation clusters.
2. If the differences were similar (+/- 1%) we selected the *k* that maximized the average SSC.

The choice of a stringent cutoff of 1% to prefer two clusters over three if the two cluster structure provides a better SSC was justified by the fact breaking observations into more clusters is not likely to make the results much worse, while under-segmenting has a potential to blur the further analysis.

#### Random Forest procedure

We calculated the 10-fold cross-validated adjusted balanced accuracy (ABAC) with balanced class weights using 500 estimators. The balanced accuracy is calculated as follows:

$$BAC=\frac{1}{\sum_{i} w_{i}}\sum_{l} \mathbf{1}(\hat{y}_{l}=y_{l})\hat{w}_{l}$$

with $\hat{w}_{i}= \frac{w_{i}}{\sum_{j} \boldsymbol{1}(y_{i} = y_{j})w_{j}}$ where $y_{i}$ and $\hat{y}_{i}$ are the truth and estimated (resp.) value for sample $i$ and $w_{i}$ the associated sample weight. The adjusted balanced accuracy reports the relative increase from $\frac{1}{n_{classes}}$, the value of BAC for the random case (thus $ABAC\in[-1,1]$). We selected cancer types whose classifiers performed better than random, *i.e.* with $ABAC>0$, and whose most useful features were persistently selected across folds. We represented the 10 highest ranked features picked by the classifiers for these cancer types ranking by feature importance and indicating the group (low or high-methylation) in which the mutation was enriched.

#### COAD mutational burden correction for significant mutations

To account for the difference in mutation rate between the high- and low-methylation groups of COAD, we first sought to quantify the enrichment of mutations in the high-methylation group as compared to expected by random chance. We introduced the Methylation Gene Mutation Enrichment (MGME) as follows:

$$MGME=\frac{\frac{n_{mutationsingeneofinterest,high\text{-}meth.group}+1}{n_{mutationsoverall,high\text{-}meth.group}+1}}{\frac{n_{mutationsingeneofinterest,low\text{-}meth.group}+1}{n_{mutationsoverall,low\text{-}meth.group}+1}}$$

In brief, $MGME>1$ signifies the studied gene is mutated more than expected by random chance in the high-methylation group as compared to the low-methylation group, $MGME<1$ the other way around. We introduced a pseudo-count to avoid cases where the gene of interest was not mutated in the low-methylation cluster.

We then sought to compute empirical *p*-values associated with the significance of the obtained MGME values. Let us call ${MGME}_{g}$the $MGME$ value of the gene $g$ of interest. For every gene $g$, we computed the MGME values of 500 genes with properties similar to gene $g$: ${MGME}_{random,j}$ for $j\in\{1,...,500\}$. The empirical *p*-value was computed as the fraction of times the ${MGME}_{random,j}$ were found more extreme than ${MGME}_{g}$ computed for the gene of interest, *i.e.*

$p = \sum_{j} \frac{{\boldsymbol{1}(MGME}_{random,j}>{MGME}_{g})}{500}$ if ${MGME}_{g}>1$;

$p = \sum_{j} \frac{{\boldsymbol{1}(MGME}_{random,j}<{MGME}_{g})}{500}$ if ${MGME}_{g}>1$.

We chose 500 genes with properties similar to gene $g$ to account for the fact that longer genes have a higher mutation frequency; moreover, transcription rate can also affect mutation probability due to the activity of different DNA repair mechanisms in expressed and silent genes. To select genes with properties similar to gene $g$, we represented all the genes available in the mutation dataset provided by TCGA (MC3) in a two-dimensional space represented by their length and their average expression in COAD. Then we performed standard scaling and computed the 1,000 nearest neighbors of the gene of interest. Finally, we picked the genes at random 500 times among the 1,000 nearest neighbors.

The results of the empirical *p*-value computation are indicated in the Supplemental Table 5.

#### Analysis CGI of methylation signature

To identify methylation signatures potentially linked to the CIMP status or etiology, we computed the average methylation value over shelves, shores, and CpG islands between the low-, intermediate-, and high-methylation groups and averaged beta-values of CpG probes in those regions. Specifically, we binned the north shelf (N_shelf) and north shore (N_shore), CpG island, south shelf (S_shelf) and south shore (S_shore) into 10 bins each. Of note, CGIs are of variable size causing variable sized bins while shore and shelves bins were systematically 200 bp long. We then computed the average of beta-values of CpG probes selected for clustering over each bin and plotted these averages for the different methylation groups, for each type of cancer.

Supplemental References

[109. Moore LD, Le T, Fan G. DNA methylation and its basic function. Neuropsychopharmacology 2013; 38:23–38](http://paperpile.com/b/NzlxcQ/Xj9zn)

[110. de Necochea-Campion R, Ghochikyan A, Josephs SF, et al. Expression of the epigenetic factor BORIS (CTCFL) in the human genome. J. Transl. Med. 2011; 9:213](http://paperpile.com/b/NzlxcQ/99vW8)

[111. Santoro R, Li J, Grummt I. The nucleolar remodeling complex NoRC mediates heterochromatin formation and silencing of ribosomal gene transcription. Nat. Genet. 2002; 32:393–396](http://paperpile.com/b/NzlxcQ/e3jSF)

[112. Lee GE, Kim JH, Taylor M, et al. DNA methyltransferase 1-associated protein (DMAP1) is a co-repressor that stimulates DNA methylation globally and locally at sites of double strand break repair. J. Biol. Chem. 2010; 285:37630–37640](http://paperpile.com/b/NzlxcQ/jkxU1)

[113. Bledea R, Vasudevaraja V, Patel S, et al. Functional and topographic effects on DNA methylation in IDH1/2 mutant cancers. Sci. Rep. 2019; 9:16830](http://paperpile.com/b/NzlxcQ/MjRDz)

[114. Hyun K, Jeon J, Park K, et al. Writing, erasing and reading histone lysine methylations. Exp. Mol. Med. 2017; 49:e324](http://paperpile.com/b/NzlxcQ/vCjCI)

[115. Legendre CR, Demeure MJ, Whitsett TG, et al. Pathway Implications of Aberrant Global Methylation in Adrenocortical Cancer. PLoS One 2016; 11:e0150629](http://paperpile.com/b/NzlxcQ/s6D6G)

[116. Harris RA, Nagy-Szakal D, Pedersen N, et al. Genome-wide peripheral blood leukocyte DNA methylation microarrays identified a single association with inflammatory bowel diseases. Inflamm. Bowel Dis. 2012; 18:2334–2341](http://paperpile.com/b/NzlxcQ/kdzH2)

[117. Sandoval J, Heyn H, Moran S, et al. Validation of a DNA methylation microarray for 450,000 CpG sites in the human genome. Epigenetics 2011; 6:692–702](http://paperpile.com/b/NzlxcQ/ugvzF)

[118. Weinhold L. A Statistical Model for the Analysis of Beta Values in DNA Methylation Studies. BMC Bioinformatics 2016; 17:480](http://paperpile.com/b/NzlxcQ/NWBIt)

[119. Ellrott K, Bailey MH, Saksena G, et al. Scalable Open Science Approach for Mutation Calling of Tumor Exomes Using Multiple Genomic Pipelines. Cell Syst 2018; 6:271–281.e7](http://paperpile.com/b/NzlxcQ/5AxCw)

[120. Yoshihara K, Shahmoradgoli M, Martínez E, et al. Inferring tumour purity and stromal and immune cell admixture from expression data. Nat. Commun. 2013; 4:2612](http://paperpile.com/b/NzlxcQ/v6Icf)

[121. Love MI, Huber W, Anders S. Moderated estimation of fold change and dispersion for RNA-seq data with DESeq2. Genome Biol. 2014; 15:550](http://paperpile.com/b/NzlxcQ/wc689)
